# Aerobic glycolysis drives differentiation of unilocular adipocytes

**DOI:** 10.1016/j.jlr.2026.101023

**Published:** 2026-03-17

**Authors:** Alice Maestri, Min Cai, Ruby Schipper, Julia Backman, Alana Vannay, Anneli Olsson, Ewa Ehrenborg, Roland Nilsson, Carolina E. Hagberg

**Affiliations:** Division of Cardiovascular Medicine, Department of Medicine Solna, Karolinska Institutet and the Center for Molecular Medicine, Karolinska University Hospital, Stockholm, Sweden

**Keywords:** adipocyte, lipid droplet, unilocularity, metabolism, aerobic glycolysis

## Abstract

The hallmark of white adipocytes is a single, large lipid droplet, yet this *unilocular* morphology has remained challenging to reproduce in vitro due to limited understanding of its molecular drivers. Here, we identify metabolic reprogramming as the key determinant of adipocyte unilocularity in human 3D cultures. Transcriptomic and metabolomic profiling revealed unilocularity to be primarily characterized by enhanced aerobic glycolysis and reduced mitochondrial content. Importantly, this mirrored the metabolic pattern of freshly isolated human white adipocytes but contrasted with that of preadipocytes and 2D cultures. We demonstrate that aerobic glycolysis in adipocytes activates AMP-activated protein kinase (AMPK), which then enhances CD36-mediated fatty acid uptake. Pharmacological inhibition of aerobic glycolysis reduced fatty acid uptake and led to a more multilocular phenotype, which could be rescued by reactivating AMPK. Importantly, limiting mitochondrial activity or activating AMPK in multilocular 3D cultures was enough to promote their unilocularity. These findings establish aerobic glycolysis as a key driver of white adipocyte lipid droplet morphology and size. They also underscore the influence of cellular microenvironment on shaping adipocyte metabolism and function. Taken together, the study provides important insights of adipocyte lipid droplet biology that can be used to improve human adipocyte functionality and prevent disease.

## Highlights

• Formation of a single unilocular lipid droplet requires aerobic glycolysis.
• This reflects the metabolism of freshly isolated human adipocytes.
• Aerobic glycolysis activated AMPK and increased adipocyte fatty acid uptake.
• Inhibition of either aerobic glycolysis or fatty acid uptake reduces adipocyte locularity.
• 2D adipocyte cultures display high basal activation of inflammation and fibrosis.

## Graphical Abstract

**Figure:**
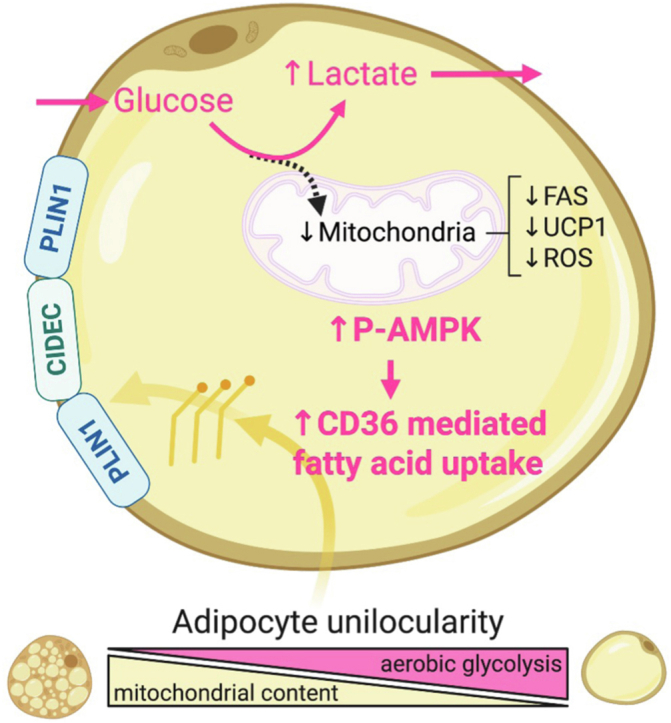

The global rise in obesity continues to fuel the incidence of metabolic diseases such as type 2 diabetes, steatotic liver disease, and dyslipidemia (1). Advances in genetics and molecular biology have redefined our understanding of these conditions, implicating insufficient lipid storage capacity as a key pathogenic driver, and positioning white adipocytes as an early, causative cell type (1, 2). Indeed, maintained lipid storage capacity within human subcutaneous adipose tissue in obesity is strongly associated with improved metabolic profiles and reduced risk of cardiometabolic disease (2, 3). These observations have intensified efforts to delineate the pathways that govern adipocyte lipid storage and functionality in health and disease in humans. As much of the mechanistic insights into human adipocyte biology are being gained through in vitro work, this also puts new emphasis on understanding the model systems we use (4).

While no in vitro system fully recapitulates the complexity of white adipose tissue, two-dimensional (2D) culture models have to date been instrumental in advancing our understanding of adipocyte lipid storage (5). However, 2D-differentiated adipocytes, including those stemming from primary adipocyte progenitors (pre-adipocytes), acquire in culture beige adipocyte-like characteristics such as low expression of uncoupling protein 1 (UCP1) (5), potentially due to the frequent use of thiazolidinediones in adipogenic differentiation media (6). In addition, all 2D adipocyte models are inherently *multilocular*, characterized by the presence of multiple, small lipid droplets in each cell. As a result, they lack the key morphological feature of white adipocytes: a single, large, central lipid droplet, termed unilocularity. This distinction is important, as lipid droplet number and size (i.e., locularity) influence the surface area-to-volume ratio of the lipid droplets, with potential implications for the adipocytes’ lipid turnover dynamics and storage capacity (7, 8, 9). 2D-cultured adipocytes are also functionally different from their in vivo counterparts as they use glucose to *de-novo* synthesize a large part of their stored fatty acids through the action of fatty acid synthase (FAS) (10). Notably, this pathway is dependent on glucose flux through the mitochondria, with mitochondrial conversion of glucose to citrate being an obligatory step prior to fatty acid synthesis by FAS and other enzymes in the cytoplasm. This makes 2D cultured adipocytes dependent on mitochondrial oxidative metabolism thought out their lifespan (11, 12). In contrast, *de-novo* synthesis of fatty acids by white adipocytes in vivo is very low (13, 14, 15), and instead most fatty acids are taken up from the surrounding microenvironment by fatty acid transporters such as CD36 (16). Together, the morphological and functional differences between white adipocytes in vivo and in 2D cultures suggest 2D cultures may not always be the optimal model system for studying human white adipocyte lipid dynamics.

To address this, our laboratory recently developed a three-dimensional (3D) adipocyte culture model that is scaffolded by growth-factor reduced Matrigel and thereby achieves differentiation of unilocular adipocytes in vitro, termed Human Unilocular Vascularized Adipocyte Spheroids (HUVAS) (4, 17). We could show that the HUVAS model displays a higher degree of unilocular adipocytes and significantly larger lipid droplets than standard 2D or scaffold-free 3D cultures (17), but the underlying cellular pathways driving these morphological features were not investigated and remain unknown. Seminal studies by others have shown Cell Death Inducing DFFA Like Effector C (CIDEC, also known as FSP27) to play a key role for adipocyte unilocularity by facilitating lipid transfer between droplets and thereby promoting lipid droplet fusion (18). Impaired CIDEC activity leads to the formation of white multilocular adipocytes, and causes lipodystrophy, hepatic steatosis and insulin resistance in both mice and humans, highlighting the physiological importance of adipocyte unilocularity (9, 19). Several regulatory mechanisms of CIDEC activity and lipid droplet size have also recently been identified (20, 21, 22, 23), including Perlipin 1 (PLIN1), the major lipid droplet associated protein of white adipocytes (23, 24). Although most of these studies have used 2D cultured multilocular cells and mainly showed increased lipid droplet size, overexpression of both CIDEC and PLIN1 has been shown to be enough to form unilocular 2D cultured human adipocytes (24). This raises the question if pathways exist that can be used to promote unilocularity and lipid storage in human white adipocytes. We hypothesized that culturing our unilocular HUVAS adipocytes side by side with isogenic multilocular adipocytes stemming from the same donors would offer a unique opportunity to identify cellular programs that boost adipocyte unilocularity in vitro.

Here we show that metabolic reprogramming toward aerobic glycolysis is a defining feature of *in vitro*-differentiated unilocular adipocytes that increases lipid droplet size by activating AMPK and CD36-mediated fatty acid uptake. Inhibiting either aerobic glycolysis or fatty acid uptake reduces lipid droplet size and unilocularity, while promoting lactate production in multilocular 3D cultured cells is enough to increase unilocularity. Furthermore, we show that 3D cultured adipocytes express less UCP1 and fewer inflammatory transcripts than isogenic 2D cultures, demonstrating how culture environment influences not only metabolism but multiple important aspects of adipocyte biology. Taken together, our results highlight the intricate crosstalk between culture conditions, metabolism and adipocyte lipid droplet dynamics. They will thereby significantly advance our understanding of both currently available culture models, and the pathways that promote human adipocyte lipid storage in metabolic health and disease.

## Materials and Methods

### 2D, 3D, and HUVAS cultures including inhibitor treatments

All adipocyte cultures used the same commercially available subcutaneous stromal vascular fraction (SVF) cells obtained from healthy donors with BMI 25–28 (Lonza, PT5001) and passaged two times before the start of the cultures. For HUVAS and 3D spheroids cultures, 10.000 SVF cells were seeded each well in 96-well ultra-low attachment plates (Corning, Costar CLS7007) and incubated at 37°C with 5% CO_2_ as previously described (17). From plating day (d)0 to d10, all cells were cultured in 200 μl/well of endothelial growth medium (EGM-2, Lonza CC- 3162) to uphold endothelial viability. To promote the formation of one single spheroid per well, cells were pipetted up and down on d3. On day d6, HUVAS but not 3D cultures were imbedded in 40 μl of growth-factor-reduced Matrigel (Corning, ref 356,231). The Matrigel (scaffold) imbedding is thereby the only difference between HUVAS and 3D cultures but allows endothelial cells to sprout and form a differentiation niche in HUVAS cultures, as described before (17). On d10, half of the media in all cultures was replaced with 2x concentrated preadipocyte differentiation medium (PGM-2, Lonza PT-8002), followed by 1x PGM-2 supplementation on days 15 and 25, and a medium exchange on day 20. All cultures were allowed to differentiate for 20 days (d10-d30) and maintained as described until harvest and analysis on d30. Note that the adipogenic PGM-2 differentiation media does not contain thiazolidinediones (as communicated by Lonza). Pharmacological activation or inhibition of spheroids used 1 mM dimethyloxalylglycine (DMOG, Merck, d20-30); 10 μM PX-478 (Sigma, d20-30); 1 mM phenylbutyrate (PB, Sigma-Aldrich, d10-30); 25 mM sodium oxamate (SO, Merck, d10-30); 5 nM rotenone (Rot., Merck, d10-20); 10 μM sodium azide (NaN_3_, VWR, d10-20) (later inhibition of mitochondrial function (d20-d30) with rotenone or sodium azide led to a lack of maturation); 200 μM CD36 inhibitor (SMS121, MedChemExpress, d20-30); 0,5 mM Metformin (SelleckChem, d10-d30 or d20-d30); 5 μM O-304 (MedChemExpress, d20-d30); 1 μM MK8722 (MK, MedChemExpress, d20-d30); and 10 μM Compound C (MedChemExpress, d10-d30 or d20-d30).

In parallel to the spheroid cultures, SVF cells from the same donor were seeded as 2D cultures onto imaging wells (Nunc Lab-Tek 4-well Chamber Slides, no. C6932 Merck) or 24-well plates (Corning, #353047) at 30k cells/cm^2^ using the same EGM2 media as for spheroids during seeding and expansion (Lonza) with the same media to cell ratio. Instead of the initial 10 days of spheroid formation, 2D cells were allowed to reach confluency (approx. 3–5 days) before differentiation was started by changing half the media to 2x concentrated preadipocyte differentiation media (2xPGM2, Lonza). Media supplementation on d15, d20 and d25 was done similarly as for the HUVAS and 3D cultures described above, as well as 20 days of adipocyte differentiation (d10–30).

For all three cultures (HUVAS, scaffold-free 3D spheroids and traditional 2D cultures), the fully differentiated adipocytes on d30 were washed with DPBS and either used for functional assays (see below), protein lysate, RNA preparation or fixed 30 min with 10% formalin, stained and imaged, and media was collected for metabolomics. To compare secreted factors between the methods, measurements were normalized to DNA or protein content as described below.

### Lipid staining and quantification of lipid droplet size and locularity

Adipocyte spheroids were collected on d30, incubated in pre-chilled cell recovery solution (Corning, #354253) at 4°C for 20 min to remove the Matrigel, fixed in 10% formalin for 30 min, and then stained with CellMask (1:500, Invitrogen, C10046), 1:2000 Bodipy-FL (1 mg/ml, D3822, Invitrogen), and DAPI (final concentration 2 μg/ml, D1306, Thermo Scientific) diluted in PBS-0.1% Tween at room temperature overnight. After staining, spheroids were washed twice for 5 min in DPBS and then mounted on microscopy slides using iSpacers (#IS010, SunJin Lab Co.) and 90% glycerol as mounting medium. Immunofluorescence images were taken with a Nikon Ti-2E confocal microscope using NIS Elements software. Adipocyte area and the diameter of the single largest lipid droplet in each cell (to obtain representative, correctly weighted data from a mixture of multilocular, paucilocular and unilocular adipocytes) were measured from the confocal images using the Fiji ImageJ software. Lipid droplet area was calculated from the lipid droplet diameter (d) using the formula A = πd^2^/4. A minimum of 50 cells were measured per spheroid. Unilocularity (for each individual adipocyte) was calculated as the ratio of its lipid droplet area over total adipocyte area (representing 100%) and analyzed using ImageJ. Post-analysis of lipid droplet size was performed in GraphPad Prism. Total spheroid area was analyzed from light microscopy pictures using an EVOS microscope and measured using the Fiji ImageJ software.

### Capillary Western blot

Capillary Western blot was used to detect the protein expression in adipocyte spheroids and 2D cultures as single spheroid samples are too small for traditional Western blot. Each spheroid was homogenized in 50 μl of radio-immunoprecipitation assay (RIPA) buffer, while 250 μl was used for 2D samples that contained 5x more cells. Lysates were processed using 5 mm stainless steel beads (Qiagen, 69989) and a QIAGEN Tissue Lyser at 30 Hz for 3 min, and centrifuged at 4°C at 16,000*g* for 15 min, and the supernatant was collected for capillary Western blot analysis. 3 μl sample of supernatant protein (100 μg/ml) was premixed with master mix and 10 μl of primary antibody was loaded onto the Jess ProteinSimple system (Biotechne, SM-W004), where capillary electrophoresis and immunodetection were performed automatically using secondary antibodies provided by the manufacturer, all according to the manufacturer’s guidelines. Protein quantification was performed using the Jess Simple Western system (ProteinSimple/Bio-Techne). Total Protein detection was carried out using the Total Protein Detection Module (Biotechne, DM-TP01) for normalization. All data processing and signal quantification were performed in Compass for Simple Western software (Version 6.1.0). Electropherogram peak areas for immunoblots were quantified within the user-defined Analysis Range Region. For discrete, non-overlapping immunoblot peaks, signal integration was performed using the Gaussian distribution fitting option. For Total Protein assays or in cases where immunoblots exhibited overlapping peaks, the Dropped Lines integration mode was applied, in accordance with the manufacturer’s recommendations. Pseudo-blot images were generated using the High Dynamic Range (HDR) 4.0 exposure setting, which, per software documentation, combines information from multiple exposures to improve signal-to-noise performance while preserving linearity at higher protein concentrations. The Baseline option was kept on during visualization to maintain consistent background correction across samples. For samples that produced diffuse bands, pseudo-blot visualization was taken from the Full Region display mode to avoid altering the predefined analysis boundaries, ensuring that quantification remained unaffected. Immunoblotting was performed using the following primary antibodies against: PLIN1 (1:10, PA5-81240, Fisher Scientific), CIDEC (1:10, PA1-46128, Invitrogen), UCP1 (1:50, MAB6158, R&D Systems), PPARγ (1:50, 2435S, Cell Signaling), FAS (1:50, 3180, BioTechne), ATGL (1:50, 2138, Cell Signaling), Total OXPHOS cocktail (1:50, Ab110411, Abcam), TOMM20 (1:50, NBP1-81556, BioTechne), PTP1B (1:50, Ab244207, Abcam), HIF1α (1:100, NBP2-75977, Novus), P-AMPK (Thr172, 1:10, 2535, Cell Signaling), total AMPK (1:50, 2532, Cell Signaling), P-HSL (Ser660, 1:10, 45,804, Cell Signaling), P-HSL (Ser563, 1:10, 4139, Cell Signaling), total HSL (1:50, 4107, Cell Signaling), and CD36 (1:50, AF1955, R&D Systems).

### RNA extraction and qPCR analysis of 3D spheroids and HUVAS

Single spheroids were harvested every 5 days of the culture protocol and lysed in 350 uL QIAzol (Qiagen, 79,306) with a 5 mm stainless steel bead, using a QIAGEN Tissue Lyser at 30 Hz for 3 min. RNA was extracted with chloroform and the PicoPure RNA Isolation Kit (Applied Biosystems, KIT0204), eluted in RNase-free water, and quantified with a NanoDrop. 200–250 ng RNA was reverse transcribed using the High-Capacity RNA-to-cDNA kit (Applied Biosystems, 4387406). qPCR was performed in duplicates using the StepOnePlus Real-Time PCR System (Applied Biosystems) with TaqMan Fast Advanced Master Mix and MicroAmp Fast Optical 96-Well Plates (Applied Biosystems, 4444556 and 4346906). TaqMan primers for human *PDGFRA* (Hs00998018_m1), *CEBPA* (Hs00269972_s1), *PPARG* (Hs01115513_m1), *PLIN1* (Hs00160173_m1), *CIDEC* (Hs01032998_m1) and *B2M* (Hs00187842_m1) were used. Relative expression was calculated using the 2ˆΔCt method with *B2M* as the reference gene.

### Bulk mRNA sequencing

For transcriptional analysis, 9 HUVAS or 3D spheroids were pooled for each technical replicate, while technical replicates for 2D adipocytes consisted of all cells from one imaging well. Cells were homogenized in Trizol using 5 mm stainless steel beads (Qiagen, 69989) and a QIAGEN Tissue Lyser at 30 Hz for 3 min whereafter chloroform was added to separate the aqueous and organic phases. Total RNA was subsequently extracted using the RNeasy Micro kit (74004, Qiagen) according to manufacturer’s protocol. The RNA concentration was assessed using NanoDrop and integrity using BioAnalyzer. Only samples with RIN > 8 were included in analysis and sent to Novogene, for sequencing and library preparation. RNA was selected using a Poly(A) RNA Selection Kit (Lexogen), and sequencing libraries were prepared with Lexogen QuantSeq version 2. DNA fragments 200–800 bp for RNAseq were selected. Cluster generation and sequencing was carried out by using an Illumina HiSeq version 4 system with a read length of 50 nucleotides (single-read) or NovaSeq with a read length of 150 nucleotides (paired-end) and aligned by HISAT2 to human genome assembly version of GRCh38/hg38. Reads per gene were counted using HTSeq with the overlap resolution mode set to union.

Differential expression of mRNA was analyzed using DESeq2 software at default settings, with a false discovery rate set at 0.01. Pathway analysis was performed for functional annotation of a gene list using an integrated functional database (clusterProfiler). Graphical representation (PCA plot and heatmaps) was performed using variance stabilizing transformation (vst). For the visualization of common differentially expressed genes (DEGs) among 2D, 3D and HUVAS, we excluded all genes not common in the other lists and with padj >0.001. Genes related to inflammation, collagen production, and markers for structural Wnt-regulated adipose tissue-resident (SWAT) cells (25) were extracted for visualization (vst in heatmap). The sequence data generated in this study were integrated with available RNA-Seq data from non-cultured adipose tissue, preadipocytes and mature adipocytes by Harms *et al.* ((26), GSE115020) and harmonized the sequencing datasets using ComBat_seq to adjust batch effects (27). Differential expression of mRNA was analyzed using DESeq2 software at default settings, with a false discovery rate set at 0.01. Graphical representation (PCA plot and heatmaps) was performed using vst. Adipocytes- and preadipocytes-enriched genes (top 50) were extracted from Ehrlund *et al.* (28). Alternatively, the HUVAS RNA-seq data was integrated with the publicly available dataset of subcutaneous adipose tissue from lean, healthy obese, and unhealthy obese subjects from Cifarelli *et al.* 2020 ((29); GSE152991) and harmonized using ComBat_seq to adjust batch effects (27). Differential expression of mRNA was analyzed using DESeq2 software at default settings, with a false discovery rate set at 0.01. Graphical representation (PCA plot and heatmaps) was performed using vst. Condition centroids were calculated as the mean PCA coordinates of biological replicates, and Euclidean distances between centroids were used to quantify transcriptional differences between conditions.

**Fig. 1:**
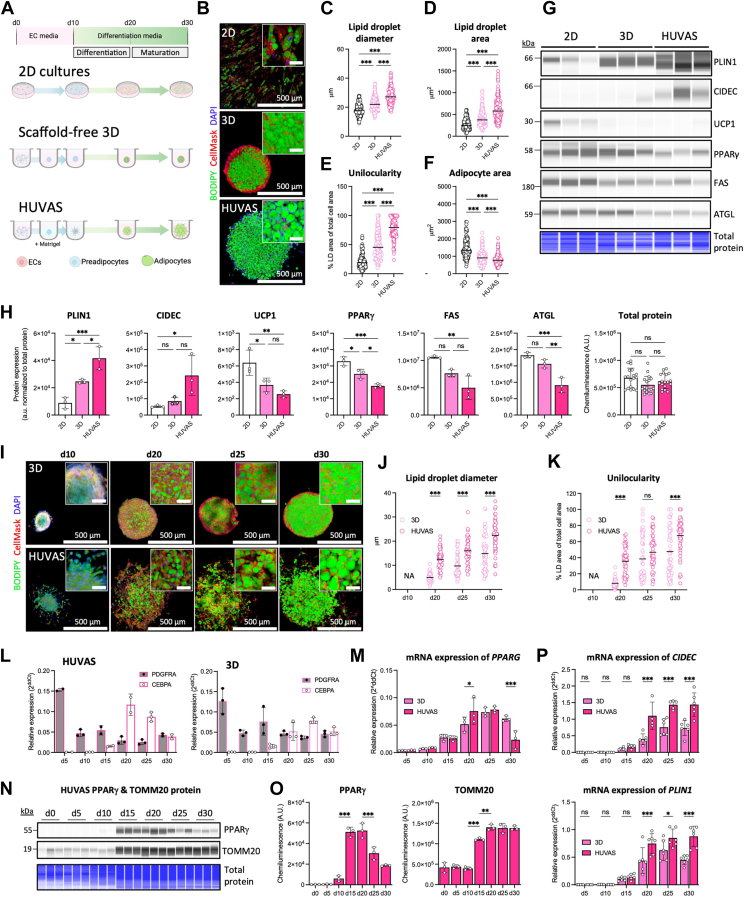
The HUVAS culture model facilitates the differentiation of unilocular human white adipocytes in vitro. A: Schematic of 2D, scaffold-free 3D, and scaffolded HUVAS cultures, done side by side using stromal vascular fraction cells from same donor and passage, cultured in the same media and with the same differentiation time, varying only the cell environment. B: Representative confocal images of BODIPY, CellMask, and DAPI-stained adipocytes on day (d)30. Scale bars for all images in Figure 1: 500 μm (main), 50 μm (inset). C–F: Quantification of lipid droplet diameter (C), total lipid droplet area (D), adipocyte unilocularity (E), and total adipocyte area (F) across isogenic 2D, 3D, and HUVAS cultures from n = 50 cells per spheroid and 3 spheroids per individual. Similar results were obtained from cells from three different cell donors. G, H: Capillary Western blot analysis on d30 of PLIN1, CIDEC, UCP1, PPARγ, FAS and ATGL protein and quantification normalized to total loaded protein (UCP1 blot shown here, all original blots shown in supplemental Fig. S1A,) from 3 technical replicates of the same individual. All total protein measurements (n = 3 per sample x 6 proteins of interest, n_tot_ = 18) are shown in H (right panel). I: Representative confocal images of BODIPY, CellMask, and DAPI-stained 3D and HUVAS spheroids on d10, d20, d25 and d30. J, K: Quantification of lipid droplet diameter (J) and adipocyte unilocularity (K) across timepoints from n = 50 cells from one spheroid per condition, repeated at least two times. L: Relative mRNA expression levels of preadipocyte marker *PDGFRA* and adipogenesis marker *CEBPA* at d5, d10, d15, d20, d25 and d30 in HUVAS (left) and 3D (right) assessed by qPCR from 3 spheroids of the same individual and repeated twice with similar results. M: Relative mRNA expression of *PPARG* across timepoints in 3D compared to HUVAS analyzed as in L. N, O: Capillary Western blot for PPARγ and TOMM20 across timepoints, with quantification shown as raw chemiluminescence values by loading equal *amounts* of spheroid lysate, instead of equal protein amount, for each timepoint from 3 spheroids (technical replicates) of the same individual. P: mRNA expression levels of *CIDEC* and *PLIN1* at d5, d10, d15, d20, d25 and d30 assessed by qPCR from >3 technical replicates of two different individuals. Mean and standard deviation (SD) shown in all applicable graphs throughout the paper unless specifically indicated otherwise.

2D, 3D, and HUVAS gene expression levels were correlated with lipid droplet size using Pearson correlation. To identify genes whose expression correlates with lipid droplet size, we first selected the set of differentially expressed genes (DEGs) common across our three conditions. For these DEGs, we calculated Pearson correlation coefficients between gene expression (log2-transformed counts) and utilized the lipid droplet size measurements from Figure 1C (averaging the LD size of 300 cells coming from 3 independent spheroids). A correlation coefficient threshold of r > 0.7 and a Benjamini-Hochberg adjusted *P*-value (FDR) < 0.1 was applied to select significantly correlated genes. These genes were then subjected to Gene Ontology (GO) enrichment analysis using the clusterProfiler R package, considering all DEGs genes as background (Biological Process (BP) ontology). GO terms were adjusted for multiple testing using the Benjamini-Hochberg method, and significantly enriched terms (FDR < 0.05) were reported. To further investigate functional associations, all significantly correlated genes were put into the STRING database. Non-connected genes were removed, and the interaction confidence threshold was set to 0.900. The remaining network was clustered using the legacy k-means algorithm implemented in STRING, resulting in three clusters. The first cluster, which was visualized, corresponds to genes enriched in glycolysis and related metabolic processes, consistent with the GO enrichment results.

### Mass spectrometry based targeted metabolomics

LC-MS/MS analyses were performed at the Small Molecule Mass Spectrometry Core Facility, Karolinska Institutet, which receives funding from the Infrastructure Board at Karolinska Institutet. Briefly, 50 μl of media from HUVAS, 3D and 2D cultures were diluted with 250 μl of ice-cold LC-MS methanol and sonicated for 15 min (Sweep mode) in an ultrasound bath on ice. Media samples were centrifuged at 12,000 g for 15 min (8°C), then 150 μl was transferred to an LC-MC vial equipped with 300 μl insert for polar platforms. Water was extracted as blank using the same protocol; 30 μl of each supernatant was pooled and mixed by vortexing, then 3 × 150 μl were used as quality control of injection. Results are shown as relative area under the curve normalized to 2D, after subtracting media without cells (as blank measurement) and normalized to total RNA that was shown to be equal for all cultures.

### Seahorse analyses

Mitochondrial respiration and media acidification measurements were performed using the Agilent Seahorse XFe96 Analyser (Agilent Technologies), following the manufacturer’s instructions. For the assay, smaller HUVAS and 3D spheroids consisting of only 7.500 SVF cells were used, and results confirmed these look the same as spheroids with 10.000 SVF cells. The day prior to the assay, the calibration cartridge was incubated with Agilent Seahorse XF Calibrant solution (100840-000, Agilent Technologies) overnight at 37°C in a CO_2_ humidified incubator. The HUVAS and 3D spheroids were also removed from their culture plates and placed in a 96-well plate with fresh media overnight. On the day of the assay, Agilent Seahorse XFe96 Spheroid Microplate (102978-100, Agilent Technologies) was coated with Cell-Tak (354240, Corning®) as per manufacturer’s instruction, prior to the attachment of the spheroids to the bottom of the plate. Seahorse media (Seahorse DMEM media # supplemented with 25 mM glucose, 1 mM Glutamax, 1 mM pyruvate, 2% FFA-BSA, 2% FBS, 7.4 pH) was added to the Spheroid Microplate 1 h prior to the assay for equilibration in a CO_2_ humidified incubator. For the Mito Stress Test, injections were prepared to reach the final concentrations of 10 μM oligomycin, 10 μM FCCP, 10 μM R/A. For the Mito Fuel test with etomoxir, L-carnitine was added to the Seahorse media at 0.5 mM; etomoxir injection was at 40 μM. For the Mito Fuel test for pyruvate, UK-5099 was injected to a final concentration of 10 μM. For the Mito Fuel test with BPTES/Glutamax, half of the plate had Seahorse media without Glutamine and received a Glutamax injection for a final well concentration of 1 mM; the other half had a final Glutamax concentration of 1 mM and received a BPTES injection at 10 μM. All results were normalized to the average DNA content (ng per spheroid) quantified by Qubit 1X dsDNA High Sensitivity (HS) (Q33231, Thermo Fisher Scientific). Mitochondrial respiration is expressed in OCR (pmol O_2_/min/ng DNA per spheroid) or OCR % where stated.

### VEGF-A ELISA

Secreted Vascular Endothelial Growth Factor A (VEGF-A) levels were measured from culture media collected on day 30 using the VEGF Human ELISA kit (KHG0111, ThermoFisher scientific), following the manufacturer’s instructions.

### Media lactate assay

Secreted lactate levels were measured from media collected on day 5, 10, 15, 20, 25 and 30 using the L-Lactate Assay Kit (Colorimetric) (ab65331, Abcam), following the manufacturer’s instructions. Basal lactate levels were determined from fresh preadipocyte differentiation medium (PGM-2, Lonza PT-8002). OD was measured using a microplate reader (BMG LABTECH) at 450 nm. Final secreted lactate levels were calculated by subtracting basal lactate levels from the lactate levels measured at day 15, 20, 25 and 30.

### Media NEFA quantification

The levels of non-esterified fatty acids (NEFAs) in the media were measured from media collected on day 30 using the WAKO NEFA (HR) set (Reagent 1: 434-91795, Reagent 2: 436-91995, FUJIFILM Wako Chemicals Europe GmbH). A standard curve was constructed using the WAKO NEFA standard (NEFA 270-77000). 50 uL of media was used and reactions were performed by adding 100 uL reagent 1 and reading blank absorbance, followed by addition of 50 uL reagent 2 and incubation for 10 min at 37°C. OD was measured using a microplate reader (BMG LABTECH) at 550 and 660 nm. Blank media levels were subtracted from sample levels, then expressed as % NEFA left in the media.

### Lipolysis assay

To measure basal and isoprenaline-induced hydrolysis of triglycerides (lipolysis), each spheroid or 2D culture was washed and subsequently incubated for 3 h in 150ul (for spheroids) or 600ul (for 2D cells) of Krebs-Ringer buffer (136 mM NaCl, 1 mM NaH2PO4, 1 mM CaCl2, 4.7 mM KCl, 1 mM MgSO4, 1 mg/ml glucose, 25 mM HEPES, 2% fatty acid free BSA, pH 7.4) either alone (=basal), or together with 10 μM isoprenaline, a non-specific adrenergic receptors activator, in a 37°C shaking water bath incubator at 160 rpm with lids open. Secreted glycerol levels were measured in the buffer by mixing 10 μl of sample with 100 μl of Free glycerol reagent (F6428, Sigma) mixed with Amplex UltraRed Reagant (A36006, Invitrogen) at 1:100 and incubating for 15 min at room temperature. The Sigma G7795 standard solution (0.026 mg/ml) was used for standards and fluorescence intensity was measured using a fluorescence microplate reader (BMG LABTECH) at Ex/Em 530/590 nm. The readout was normalized by cell number.

### Fatty acid uptake

Adipocyte cultures were assessed for fatty acid (FA) uptake by incubating non-fixed adipocytes with 10 μM BODIPY™-C12 (C3835, Invitrogen) bound to 0.1% fatty-acid free BSA in DPBS at 37°C for 60 min, followed by lysing the cells using 5 mm stainless steel beads (69989, Qiagen) and a QIAGEN Tissue Lyser at 30 Hz for 3 min. The fluorescence intensity of the adipocyte lysate was measured using a fluorescence microplate reader (BMG LABTECH), with wavelengths adjusted according to the specific dye used. A serial dilution of the dye was performed to generate a standard curve for quantifying the fluorescence signal of each sample. Lysates without tracer-incubation were used as controls for endogenous autofluorescence, which was low compared to tracer uptake. The same lysate was used for DNA quantification using the Qubit dsDNA High Sensitivity Assay Kit (Q33231, Invitrogen) with an appropriate standard curve and used to normalize uptake measurements. For CD36-mediated FA uptake, we preincubated HUVAS and 3D spheroids with 200 μM of CD36 inhibitor SMS121 (HY-163541, MCE) for 24 h, then performed the FA uptake protocol as described above in the absence or presence of SMS121 (200 μM, HY-163541, MCE).

### Statistics

Statistical analysis was performed using the GraphPad software (Prism 9) using unpaired student's two-tailed t-tests, Kruskal-Wallis test for non-parametric dataset, ordinary one-way ANOVA with Šidák’s multiple comparison test or 2way ANOVA with Šidák’s multiple comparison test, Pearson correlation (∗*P* < 0.05, ∗∗*P* < 0.01, ∗∗∗*P* < 0.001, ns = not significant). Numerical values for each measurement are displayed as mean ± standard deviation (SD) unless stated otherwise in the figure legend.

## Results

### The HUVAS model facilitates differentiation of unilocular white adipocytes

To reconfirm that HUVAS produces unilocular adipocytes with larger lipid droplets we cultured the same commercially available human primary stromal vascular cells (from Lonza, including preadipocytes and endothelial cells but not immune cells) either as traditional 2D cultures (termed 2D), scaffold-free 3D spheroids (termed 3D), or as scaffolded HUVAS spheroids embedded in growth-factor reduced Matrigel (17), using otherwise the exact same culture settings for all three cultures conditions (Fig. 1A). This protocol uses endothelial cell media for all cultures during the initial plating and expansion phase (culture day (d) 0–10) in contrast to traditional in vitro differentiation protocols for adipocytes. All cultures were differentiated for 20 days (d10-d30) to adapt to the optimized HUVAS protocol, which is longer than the standard in the field (Fig. 1A). As a result of the longer differentiation time, the 2D cultures presented here display relatively higher adipogenic differentiation efficiency and larger lipid droplet sizes than typically reported, with the average diameter of the largest lipid droplet in each 2D adipocyte measuring 18.6 μm (Fig. 1B, C). As previously reported (17), culturing adipocytes as HUVAS achieved differentiation of significantly larger adipocyte lipid droplets and a higher level of unilocular adipocytes than for any of the other cultures (average HUVAS lipid droplet diameter 27.6 μm), while scaffold-free 3D adipocytes displayed an intermediate lipid droplet size and unilocularity phenotype (Fig. 1B–E). Cell area was the biggest for the 2D differentiated adipocytes, as expected as they spread out on the culture plate surface instead of forming 3D spheres (Fig. 1F). To assess classical markers of white adipocyte differentiation and maturation, we performed capillary Western blot of the isogenic 2D, 3D and HUVAS cultures on d30. As expected, the unilocular HUVAS adipocytes expressed the highest level of both the white adipocyte lipid droplet protein PLIN1 and the lipid transferase CIDEC, and the lowest level of the beige/brown adipocyte marker UCP1, confirming their white adipocyte identity (Fig. 1G, H and supplemental Fig. S1, normalized to total protein levels (30)). During early adipogenic differentiation (d10-20), HUVAS adipocytes readily upregulated, and expressed high levels of the major adipogenic transcription factors CCAAT enhancer-binding protein alpha (*CEBPA*) and Peroxisome proliferator-activated receptor gamma (*PPARG*), while preadipocyte marker Platelet-Derived Growth Factor Receptor Alpha (*PDGFRA*) expression declined (Fig. 1L–O). Thereafter, during further adipocyte maturation (d20-30) both *CEBPA* and *PPARG* expression reduced, and PPARγ protein levels stabilized at a lower level, yet still significantly (>40x) higher than for non-differentiated spheroids on d10 (Fig. 1L–O). At the end of the cultures, PPARγ protein expression was therefore lower in unilocular HUVAS than in multilocular 2D and 3D adipocytes (Fig. 1G, H), as were several other major lipid handling proteins such as the fatty acid synthase (FAS) and adipose triglyceride lipase (ATGL, Fig. 1G, H). Notably, *CIDEC* and *PLIN1* expression remained higher in HUVAS than in 3D spheroids throughout maturation (d20-30), with their expression correlating with observed differences in lipid droplets size but not with the emergence of unilocularity, which was manifested only at the end of the cultures on d30 (Fig. 1I–K, P). Note that unilocularity is hard to assess at d15 and d20, especially for 3D spheroids that have very small lipid droplets at these timepoints. We conclude that the mode of culture (2D/3D) and culture environment ( ± scaffold) greatly influences white adipocyte protein expression and morphology, even when all other culture parameters remain the same, and that high expression of major adipogenic markers such as PPARγ is not the dominating feature of unilocular adipocytes differentiated in vitro. This prompted us to use these inter-culture differences to pinpoint the pathways that enhance adipocyte unilocularity and lipid droplet size in vitro.

### Unilocular adipocytes are characterized by high expression of glycolytic and hypoxic genes

**Fig. 2:**
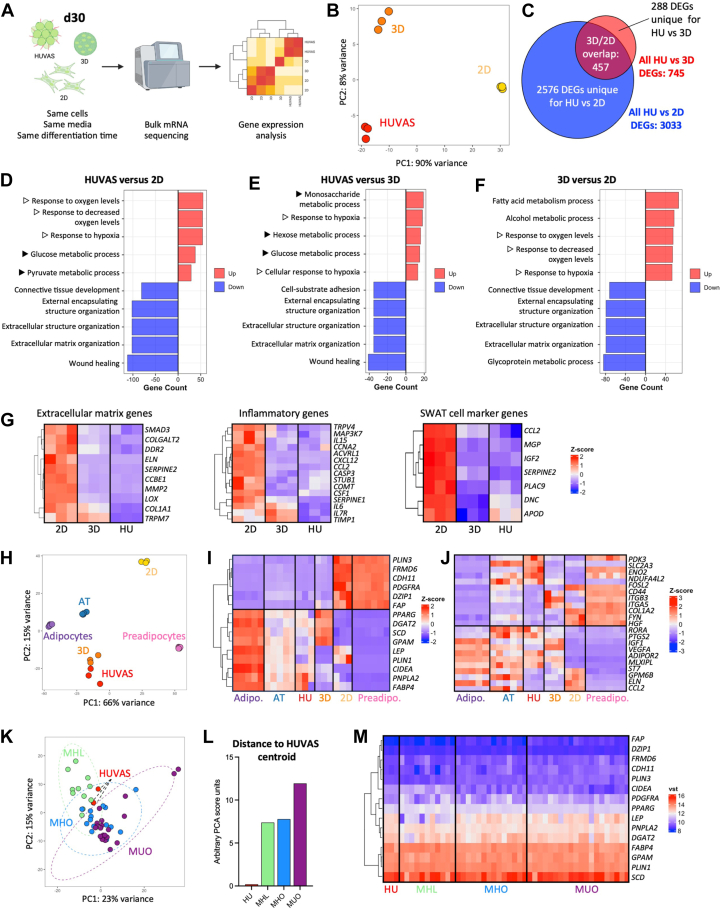
Pathway analysis reveals unilocular adipocytes to be characterized by high expression of glycolytic and hypoxia-responsive genes. A: Schematic of the RNA-Seq analytic workflow. B, C, principal component (PC) and Venn diagram for differentially expressed genes (DEGs) between the HUVAS (HU), 2D, and 3D spheroids. D–F: Top gene ontology analysis results of DEGs showing enrichment for glycolytic (filled triangle) and hypoxia (open triangle) related pathways in HUVAS compared to 2D and 3D cultures. G, relative expression heatmaps (z-scores) for genes related to extracellular matrix production (left), inflammation (middle), and SWAT cell markers (right) across cultures. H–J: PC analysis (H) and z-score heatmaps for adipocyte markers (I) and metabolic genes (J), comparing HUVAS, 3D and 2D cultures to non-cultured primary human subcutaneous adipocytes (Adipo.), adipose tissue (AT) and pre-adipocytes (Preadipo.) from (26). K–M: PC analysis (K), euclidean distance of the PCA centroids (L) and heatmap (vst) of adipocyte markers comparing HUVAS (HU) to subcutaneous adipose tissue stratified as metabolically healthy lean (MHL), metabolically healthy obese (MHO) and metabolically unhealthy obese (MHO) from (29).

To elucidate pathways driving human adipocyte unilocularity in vitro we submitted lysates from the above cultures for next generation bulk mRNA sequencing (RNA-seq), followed by gene expression and pathway analyses (Fig. 2A). Principal component (PC) analysis showed all three technical replicates for each culture condition readily clustered together, with 90% of the variance found between 2D and 3D/HUVAS cultures (represented by PC1), while the HUVAS and 3D cultures only showed 8% variance between each other (represented by PC2, Fig. 2B). The variance for each culture condition was driven by a set of unique differentially expressed genes (DEGs), with 3033 genes being differentially expressed between HUVAS and 2D cultures, while 745 DEGs were identified when comparing HUVAS to 3D cultures, despite all cultures stemming from cell of the same individual (Fig. 2C and supplemental Fig. S2A). Over 60% of the DEGs between HUVAS and 2D cultures were also different between 3D and 2D cultures (supplemental Fig. S2B, C), in line with several previous studies showing that culturing adipocytes as scaffold-free 3D spheroids greatly alters adipocytes’ phenotype as compared to 2D culturing (31, 32).

Because of the morphological differences between our HUVAS and scaffold-free 3D spheroids (Fig. 1B), we focused further studies on specifically pinpointing the pathways driving these differences. Surprisingly, Gene Ontology (GO) analyses of DEGs showed HUVAS adipocytes to mainly be characterized by increased glucose metabolism and hypoxia (indicated by filled and open triangles, respectively, in Fig. 2D, E). The DEGs included glucose transporter 3 (*SLC2A3*), phosphoglycerate kinase 1 (*PGK1*), vascular endothelial growth factor A (*VEGFA*) and pyruvate dehydrogenase kinase isoform 2 (*PDK2*) (supplemental Fig. S2D). Notably, while hypoxia was overrepresented among the top GO pathways (open triangles), hypoxia-inducible factor 1 alpha (*HIF1A*) itself and other typical HIF1α target genes were not found among the HUVAS DEGs. Compared to HUVAS, scaffold-free 3D cultures instead showed higher mRNA expression of extracellular matrix components and organization, as could be expected from their lack of an exogenously provided cell matrix scaffold (Fig. 2E in blue, and supplemental Fig. S2D). The expression of 2D cultures were completely dominated by pathways related to extracellular matrix, connective tissue and cell adhesion, as well as wound healing (Fig. 2D–G and supplemental Fig. S2D), most likely promoted by fewer cell-cell contacts and a higher need for endogenous matrix production in the 2D setting, as compared to both HUVAS and 3D cultures. In addition, 2D cultures were marked by high expression of pro-inflammatory genes and gene markers for structural Wnt-regulated adipose tissue-resident (SWAT) cells (25), which were expressed at a much lower level in HUVAS (Fig. 2G). Taken together, the sequencing analyses suggested that providing HUVAS adipocytes with an exogenous scaffold downregulated their intrinsic need for matrix production and proinflammatory gene expression, while promoting a transcriptional shift toward increased glucose utilization.

To understand if the glycolytic transcription pattern of HUVAS represented a physiological adipocyte phenotype, we integrated our sequencing data with publicly available data of primary, freshly isolated subcutaneous adipocytes, adipose tissue (AT) and preadipocytes (26), similar to what has been done previously for other 3D models (31) (supplemental Fig. S3A). Renewed PC analyses showed as expected that all three cultures from this study (2D, 3D and HUVAS) represent an intermediate phenotype between non-cultured mature adipocytes and undifferentiated preadipocytes, with HUVAS and 3D spheroids clustering together (Fig. 2H). Closer analyses of the expression of adipocyte and preadipocyte markers showed HUVAS cultures to readily express most adipocyte markers at similar levels as AT (Fig. 2I and supplemental Fig. S3B). In contrast, 3D adipocytes show an intermediate phenotype between adipocyte and preadipocyte patterns, while the 2D cultures displayed a more preadipocyte-like expression pattern, possibly driven by remaining undifferentiated cells within the cultures (Fig. 2I and supplemental Fig. S3B). Similar analysis for metabolic genes confirmed high expression of most of the HUVAS-enriched metabolic and hypoxia-related DEGs in the freshly isolated mature adipocytes and AT (Fig. 2J). To further dissect if the HUVAS transcriptional pattern was most like that of healthy or dysfunctional AT, we integrated the HUVAS sequencing data with that of subcutaneous AT from metabolically healthy lean, healthy obese, or unhealthy obese individuals (29). PC analyses showed HUVAS to cluster among the samples from healthy lean and obese subjects (Fig. 2K, L), and further transcriptional analyses confirmed HUVAS to express a similar pattern of adipocyte and preadipocyte marker genes as all AT samples (Fig. 2M). We conclude that while all cultured adipocytes display transcriptional differences from their primary counterparts, both HUVAS and 3D cultures show strong transcriptional similarity with human freshly isolated adipocytes and AT, including genes associated with glycolytic metabolism. We also highlight 2D cultures to be more pro-inflammatory and progenitor-like than any of the 3D models, in line with previous reports (31).

### Lipid droplet size correlates to increased metabolic activity and lactate production in adipocytes

**Fig. 3:**
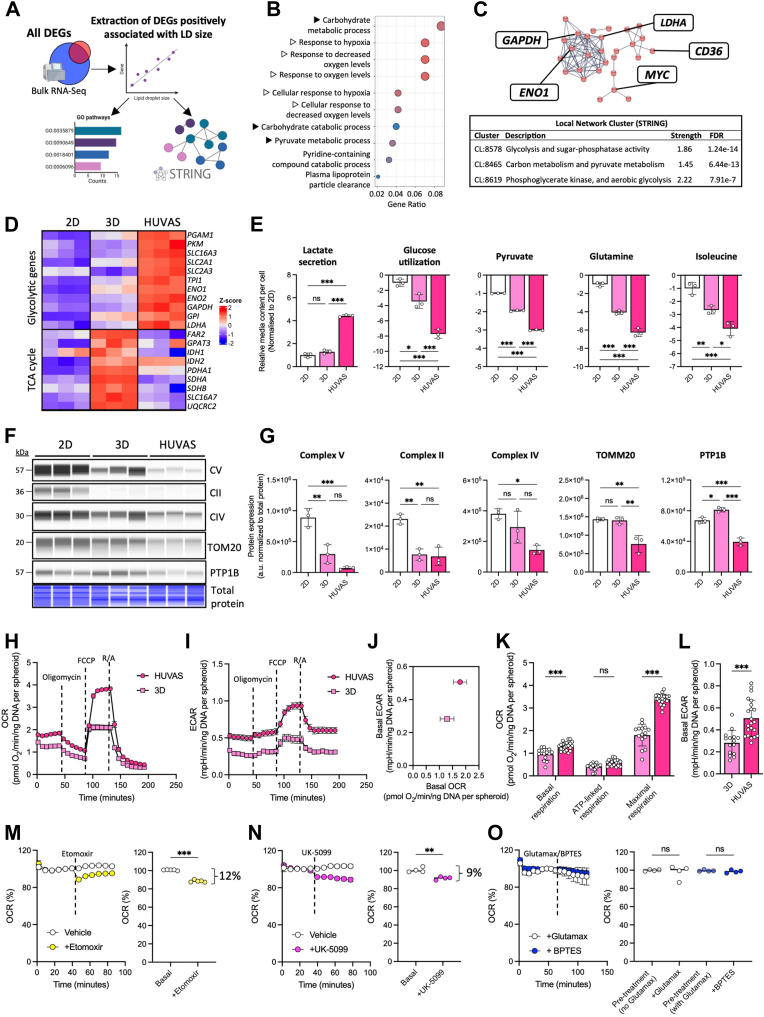
Lipid droplet size in HUVAS adipocytes correlates with aerobic glycolysis and reduced mitochondrial content. A: Workflow for RNA-seq-based identification of DEGs correlating with lipid droplet size. B-C, GO enrichment analysis (Biological Processes, B) and STRING clustering and Local Network Cluster analysis (C) of DEGs correlated to lipid droplet size across all three technical replicates from HUVAS, 3D, and 2D cultures. D, Heatmap for selected glycolytic and TCA cycle genes (z scores) across HUVAS, 3D, and 2D cultures. E: Targeted media metabolomics of the indicated metabolites across cultures from 3 spheroids from the same individual on d30. F, G, Capillary Western blot analysis of mitochondrial proteins and oxidative phosphorylation (OXPHOS) complexes, with quantification (G) after normalization to total protein (supplemental Fig. S4) from 3 technical replicates of the same individual and repeated for at least two additional donors. H–L: Seahorse assays of oxygen consumption rate (OCR, H) and extracellular acidification rate (ECAR, I) under basal and stressed conditions in HUVAS, as well as their quantification (J–L). N > 15 spheroids of the same individual per data points and repeated at least twice. Because of loss of some spheroids during analysis the Seahorse data (H–J) is shown as data in (H–J) is shown as mean and SEM. M–O, OCR changes following inhibition of mitochondrial entry of acyl-CoA (with etomoxir, M, n > 5), pyruvate (UK-5099, N, n = 4) or glutamine (BPTES, O, n > 3), with the relative change indicative of substrate preference.

We next performed a second analysis of our RNA-Seq data aimed at specifically pinpointing pathways correlating with increased adipocyte lipid droplet size. Correlating all DEGs across the 3 comparisons (n = 3846) to the average lipid droplet diameter of the nine culture replicates yielded 751 correlated genes (r > 0.7, FDR <0.1), which we subsequently characterized by GO pathway and String analyses (Fig. 3A). This analysis again highlighted glucose metabolism and hypoxia-related pathways to be the strongest characteristics of increased lipid droplet size in vitro, together with plasma lipoprotein particle clearance (Fig. 3B). Moreover, String analysis (www.string-db.org) generated 3 clusters upregulated in HUVAS, the first of which included several canonical glycolytic enzymes together with the major lactate-synthesizing enzyme lactate dehydrogenase A (*LDHA)* (Fig. 3C), and the second including the fatty acid translocase CD36 (Fig. 3C). Local Network Clustering using String also put Glycolysis, Carbon and pyruvate metabolism and Aerobic glycolysis as top 3 GO categories (Fig. 3C). Further transcriptional analysis confirmed HUVAS to express the higher level of glycolytic genes (Fig. 3D). In contrast, 3D cultures showed instead higher expression of tricarboxylic acid (TCA) cycle and oxidative phosphorylation (OXPHOS) related genes, while none of these categories of metabolic genes were overrepresented in the 2D cultures (Fig. 3D).

To validate these results, we submitted culture media from all three cultures on d30 for targeted mass spectrometry-based polar metabolomics, showing that unilocular HUVAS adipocytes secreted higher levels of lactate to the culture media, in line with their higher mRNA expression of *LDHA* (Fig. 3D, E). This was paralleled by greater uptake of glucose, pyruvate and several amino acids from the media, together confirming unilocular HUVAS adipocytes to be highly glycolytically active (Fig. 3E). In parallel, HUVAS adipocytes displayed lower protein levels of mitochondrial and OXPHOS proteins than the other cultures and reduced levels of the reactive oxygen species marker protein tyrosine phosphatase 1B (PTP1B, Fig. 3F and supplemental Fig. S4A). Monitoring HUVAS metabolism throughout differentiation showed a sharp upregulation of both lactate production and the mitochondrial marker protein translocase of outer mitochondrial membrane 20 (TOMM20) during early adipogenic differentiation (d10-20), in accordance with previous literature (33, 34), that parallelled the increase in PPARγ protein levels, cell growth and protein content of the adipocytes (Fig. 1N, O and supplemental Fig. S1B, C). However, while adipocyte mitochondrial content stabilized by d20 of culture, lactate levels in the media kept rising throughout the culture period as adipocyte lipid droplet size and unilocularity increased, suggesting the unilocular HUVAS adipocytes to be dependent on lactate production also during their later maturation phase (d20-30, Fig. 1I–K and supplemental Fig. S1B, C).

To further evaluate the metabolism of unilocular adipocytes in vitro, we performed parallel mitochondrial respiration measurements of HUVAS and 3D spheroids at the end of the cultures using a Seahorse Analyzer (Fig. 3H). Despite a trend toward lower mitochondrial OXPHOS-related protein expression (Fig. 3G), HUVAS adipocytes displayed both higher oxygen consumption and extracellular acidification rates (ECAR) (Fig. 3H, I), confirming higher metabolic activity (Fig. 3J–L). Notably, the basal ECAR was almost doubled in HUVAS as compared to 3D spheroids (+72%, *P* < 0.001), while the basal oxygen consumption rate was less increased (+33%, *P* < 0.01) (Fig. 3J–L). To better understand which substrate is oxidized by unilocular HUVAS adipocytes’ mitochondria, we performed Seahorse Mito Fuel assays blocking the mitochondrial entrance of either fatty acids, pyruvate or glutamine with etomoxir, UK-5099 and BPTES, respectively. The tests suggest a very low oxidation efficiency for both fatty acids and pyruvate (12% and 9%, Fig. 3M, N), and little oxidation of glutamine based on injections of BPTES or Glutamax (Fig. 3O). Taken together, transcriptional analysis of both DEGs and genes correlating to lipid droplet size, as well as media metabolomics and functional Seahorse analyses, all highlighted adipocyte lipid droplet size and unilocularity to correlate with a metabolic switch toward increased glycolysis and high lactate synthesis. Moreover, while HUVAS adipocytes showed reduced expression of mitochondrial proteins, Seahorse analyses confirmed the unilocular HUVAS adipocytes to be more metabolically active than the multilocular 3D cultures, a trademark of enhanced terminal cell differentiation.

### Aerobic glycolysis, rather than HIF1α activation, characterizes unilocular adipocytes

Increased lactate production can occur independent of cellular oxygenation levels as part of a metabolic program termed aerobic glycolysis (also known as the Warburg effect), but it may also indicate hypoxia and HIF1α activation (Fig. 3B). The two cellular programs can be hard to distinguish using only GO pathway analysis and media measurements, as in this case for HUVAS adipocytes (Fig. 3B, E, I). Moreover, previous studies using 2D cultures have found high lactate production to induce HIF1α stabilization and activation, initiating a feed-forward loop that promoted adipocyte lipid accumulation (35). We therefore tested if HIF1α signaling was activated in HUVAS and if this was the cause of their unilocular morphology, glycolytic metabolism and gene expression pattern.

**Fig. 4:**
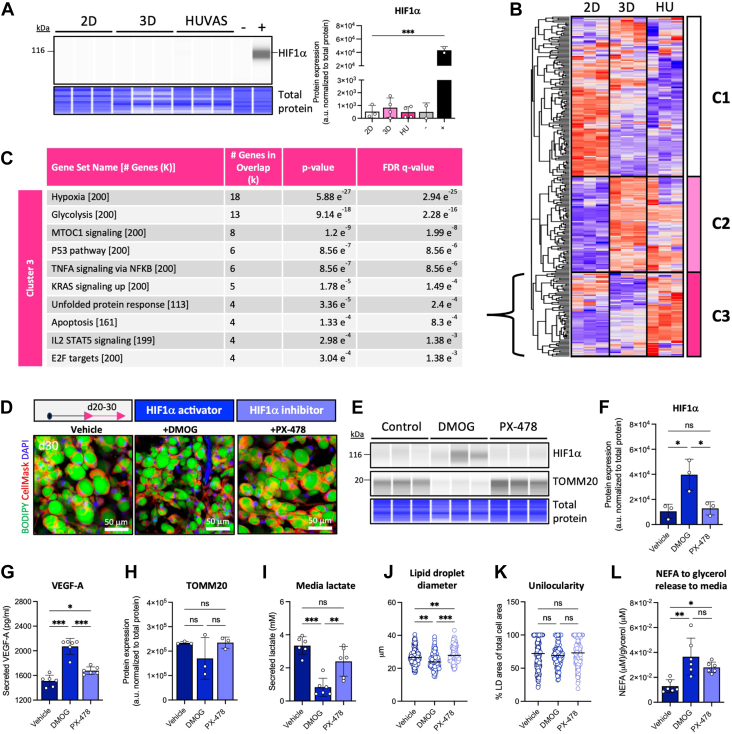
Unilocular adipocytes utilize non-oxidative glucose metabolism independent of HIF1α activation. A: Capillary Western blot analysis of HIF1α protein levels across HUVAS, 3D, and 2D cultures, with DMOG-treated endothelial cells as positive control indicated by (+), and quantification after normalization to total protein (right, supplemental Fig. S4B) from 3 technical replicates of the same individual. B, C: Sub-clustering of all hypoxia-related genes in GO:0001666 based on HUVAS, 3D and 2D RNA-seq data, showing enriched pathways for HUVAS adipocytes in C. D–L: HUVAS d30 phenotype after forced HIF1α activation (with DMOG) or inhibition (with PX-478) d20-d30, shown as representative confocal images (D), HIF1α and TOMM20 protein expression (E, F, H after total protein normalization in supplemental Fig. S4B), VEGF-A secretion to media (G), media lactate levels (I), adipocyte lipid droplet diameter (J), unilocularity (K), and the release of total non-esterified fatty acid (NEFAs) when compared to glycerol (L). All data stem from 3 to 5 technical replicates of the same individual. n = 50 cells per spheroid and 3 spheroids per individual was used for confocal image quantification. Scale bars: 50 μm.

First, we investigated if the unilocular HUVAS cultures expressed stabilized HIF1α protein, the most rigid marker of cellular hypoxia. While HIF1α protein was readily detected in control cells treated with the well-characterized pharmacological HIF1α-activator Dimethyloxalylglycine (DMOG), we failed to detect HIF1α protein stabilization in any of the HUVAS, 3D or 2D cultures, suggesting none of them were hypoxic (Fig. 4A and supplemental Fig. S4B). To assess why lipid droplet size correlated with hypoxia-related pathways (Fig. 3B), despite no discernible HIF1α activation, we used the *GO:0001666 Hypoxia* gene list to extract all hypoxia-related genes from our 2D, 3D and HUVAS RNA-Seq data (supplemental Fig. S5A). Hierarchical clustering was performed for all the GO:0001666 hypoxia-related genes and the resulting dendrogram cut into three major clusters based on expressional differences between the 2D, 3D and HUVAS cultures (Fig. 4B). Cluster hallmarks were then annotated using gene set enrichment analysis (GSEA). While all three clusters were characterized by the term *hypoxia* as per definition, cluster 1, characterizing 2D adipocytes, was also hallmarked by inflammatory pathways, and included *HIF1A* itself and other classical hypoxia-responsive genes (supplemental Fig. S5B and data not shown). In contrast, cluster 3 genes, which were most highly expressed by HUVAS adipocytes, were characterized by glycolysis and MTOC1 signaling (Fig. 4C), suggesting HUVAS unilocularity is not driven by classical hypoxic gene programs but rather associate with activated glycolysis under aerobic conditions, which shares the same metabolic GO signature as hypoxia.

To conclusively test how HIF1α activation or inhibition affects adipocyte lipid droplet size and unilocularity in 3D cultures, we treated HUVAS spheroids with the HIF1α-activator DMOG or the HIF1α-inhibitor PX-478 (Fig. 4D). Importantly, both drugs were added at non-toxic concentrations at d20 of culture, after an initial 10 days of adipogenic differentiation, allowing us to study adipocyte maturation rather than early differentiation, as HIF1α activation can impair adipogenesis (35). Successful HIF1α activation with DMOG could be verified at the end of the cultures by detecting stabilized HIF1α protein (Fig. 4E, F and supplemental Fig. S4B), by measuring higher levels of the HIF1α-downstream target VEGF-A in the culture media (Fig. 4G) and a tendency toward reduced expression of the mitochondrial marker TOMM20 (Fig. 4E, H). However, in contrast to expectations, pharmacological HIF1α activation in HUVAS significantly *reduced* the levels of secreted lactate to the media, suggesting HIF1α was not the main transcription factor driving HUVAS lactate production (Fig. 4I). Moreover, activation of HIF1α with DMOG did not increase lipid droplet size but rather reduced it (Fig. 4J) without affecting adipocyte unilocularity (Fig. 4K). The reduced lipid droplet size following DMOG treatment was most likely due to higher lipolytic activation together with decreased fatty acid re-esterification, as this has been shown previously for hypoxia-exposed 2D cultures (36) (Fig. 4L). The opposite treatment, inhibiting HIF1α activation with PX-478, had little effect on the above parameters, and notably did not reduce HUVAS lactate production, lipid droplet size, or unilocularity, suggesting HIF1α was not the driver of these phenotypes (Fig. 4H–J). Taken together, the results showed no evidence for hypoxia or HIF1α signaling to be driving the metabolic or lipid droplet profile of the unilocular HUVAS cultures. Instead, we conclude aerobic glycolysis to be a defining hallmark of in vitro differentiated unilocular human white adipocytes.

### Pharmacological manipulation of adipocyte metabolism alters lipid droplet size and unilocularity

**Fig. 5:**
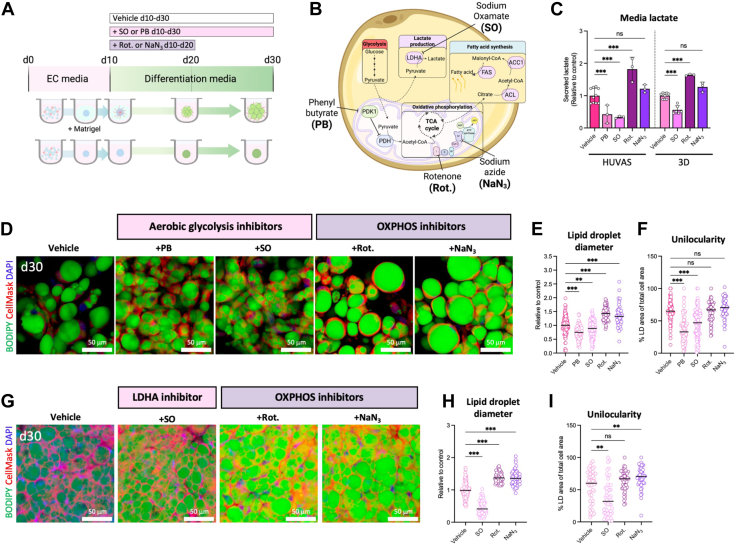
Pharmacological manipulation of adipocyte metabolism alters lipid droplet size and morphology. A, B: Schematics showing experimental workflow (A) and the pharmacological targets of aerobic glycolysis inhibitors sodium oxamate (SO) and phenylbutyrate (PB), and mitochondrial OXPHOS inhibitors Rotenone (Rot.) and sodium azide (NaN_3_). C: Adipocyte lactate secretion to the media on d30 after inhibitor treatment across HUVAS and 3D cultures, with controls normalized to the mean of the control group before combining several experiments and cell donors. n > 3 technical replicates of the same individual and at least two donors. D–I: Representative images (D, G) as well as quantification of lipid droplet diameter (E, H) and unilocularity (F, I) for HUVAS (D–F) and 3D spheroids (G–I) treated with aerobic glycolysis or OXPHOS inhibitors as described in methods. n = 50 cells per spheroid and 3 spheroids (E–F) or 2 spheroids (H–I) per individual; values in (E, F, H, and I) are expressed as relative to the mean of HUVAS or 3D control to combine data from several experiments and cell donors. Scale bars: 50 μm.

To discern if aerobic glycolysis is required for adipocyte unilocularity or only correlates with it, we set up a series of experiments using small compound inhibitors to either reduce or increase adipocyte lactate production (Fig. 5A, B). While these types of experiments have been done in 2D cultures before without reported changes in unilocularity (11, 37), they have not previously been tested on 3D-cultured adipocytes, or in the context of measuring changes in lipid droplet size and morphology. It should be noted that primary adipocytes, especially when cultured in 3D, are difficult to transfect and therefore more responsive to pharmacological inhibition. To reduce the adipocytes’ capacity for aerobic glycolysis, we used either sodium oxamate (SO (38), 25 mM) that directly binds to and inhibits LDHA, the main lactate synthase, or phenyl butyrate (PB (39), 1 mM), a PDK1-inhibitor that promotes the activity of pyruvate dehydrogenase (PDH) and thereby increases shuttling of pyruvate into the mitochondria and TCA cycle activity (Fig. 5B). Conversely, we used either the Complex I inhibitor Rotenone (5 nM), or the Complex IV inhibitor Sodium Azide (NaN_3_, 10 uM) to reduce mitochondrial OXPHOS and thereby promote pyruvate conversion to lactate (Fig. 5B, C).

Inhibiting aerobic glycolysis with either PB or SO significantly lowered the adipocytes’ lactate secretion to the media, while Rotenone increased adipocyte lactate production and NaN_3_ did not alter media lactate levels (Fig. 5C). Importantly, treating unilocular HUVAS adipocytes with either of the aerobic glycolysis inhibitors SO and PB led to the formation of adipocytes with much smaller lipid droplets and a reduced percentage of unilocular cells (Fig. 5D–F). Conversely, blocking OXPHOS activity with either Rotenone or NaN_3_ increased adipocyte lipid droplet size, but did not increase unilocularity, possibly due to the already high proportion of unilocular cells (Fig. 5D–F). These effects were not due to changes in lipid breakdown, as white adipocytes do not readily rely on beta oxidation for energy production (Fig. 3M) and HUVAS express very low levels of UCP1 (Fig. 1G). Taken together, we could show that pharmacological inhibition of adipocyte aerobic glycolysis reduces the lipid droplet size and the degree of unilocular adipocytes in vitro.

We next asked if promoting aerobic glycolysis in multilocular scaffold-free 3D cultures would be enough to increase their degree of unilocularity. 3D spheroids were therefore treated with LDHA inhibitor SO, or with OXPHOS blockers Rotenone or NaN_3_ similar to the HUVAS (Fig. 5A–C). Blocking adipocyte lactate production with SO again led to smaller lipid droplet sizes and more multilocular cells also in the 3D cultures (Fig. 5G–I). Remarkably, treating multilocular 3D adipocytes with OXPHOS blockers Rotenone or NaN_3_ led to larger lipid droplets and more unilocular adipocytes at the end of the cultures (Fig. 5G–I). It should be noted that continued inhibition of mitochondrial function throughout the culture (d10-d30) had the opposite effect and impaired adipogenesis, in line with previous results from 2D cultures (11) (not shown). Taken together, we conclude that a shift in energy metabolism toward aerobic lactate production during adipocyte maturation is enough in 3D cultures to enable the formation of more unilocular white adipocytes, causally linking adipocyte metabolism to unilocularity.

### Adipocyte AMPK-activation promotes CD36-mediated fatty acid uptake

**Fig. 6:**
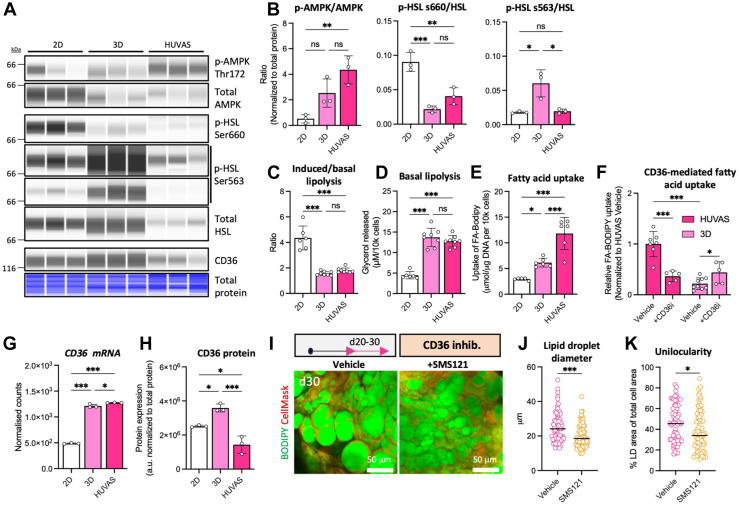
HUVAS adipocytes are characterized by AMPK activation and high fatty acid uptake. A, B: Capillary Western blot analysis of p-AMPK ^T172^, total AMPK, p-HSL^S660^, p-HSL ^S563^ (shown with 2 contrasts), total HSL, and CD36 as well as quantification of the p-AMPK/AMPK and p-HSL/HSL ratios after normalization to total protein levels (Fig. S6A, S7A) across HUVAS, 2D, and 3D cultures from 3 technical replicates of the same individual on d30. C, D, isoprenaline-induced (C, shown as induced/basal) and basal (D) lipolysis measured by glycerol release across cultures from 8 technical replicates of the same individual. Similar results were obtained from cells from >three different cell donors. E, F: Quantification of the uptake of a BODIPY-labelled long-chain fatty acid tracer (BODIPY-FA) across HUVAS, 3D, and 2D cultures (E), and in HUVAS and 3D cultures in the absence or presence of the CD36 inhibitor SMS121 or vehicle (F). N ≥ 5 throughout; values in (F) are expressed as relative to the mean of HUVAS control to combine data from several experiments and cell donors. G–H: Quantification of CD36 mRNA expression expressed as normalized counts (G) and protein from capillary Western blot analysis (H) after normalization to total protein levels (supplemental Fig. S6A) from 3 technical replicates of the same individual on d30. I–K: Effects of chronic treatment of HUVAS with CD36 inhibitor SMS121 d20-d30 shown as representative confocal images at d30 (I), adipocyte lipid droplet diameter (J), and unilocularity (K). n = 40 cells per spheroid and 2 spheroids per individual. Scale bars: 50 μm.

Finally, we wanted to understand how a metabolic shift toward aerobic glycolysis can affect adipocyte lipid droplet size and unilocularity. Reduced mitochondrial OXPHOS activity and treatment with OXPHOS inhibitor Rotenone both decrease cellular energy levels, which may activate AMP-activated protein kinase (AMPK) (40). We hypothesized that AMPK, known to modulate adipocyte lipid handling and metabolism, could be the missing link between the metabolic reprogramming and unilocularity of HUVAS. In support, we found HUVAS adipocytes to display higher levels of activating AMPK^T172^ phosphorylation as compared to 3D and 2D adipocytes (Fig. 6A, B and supplemental Figs. S6A and S7A), in line with lower expression of mitochondrial OXPHOS components in HUVAS (Fig. 3F, G). The increase in AMPK^T172^ phosphorylation in HUVAS as compared to 3D was significant only on d30 of culture, coinciding with increased HUVAS lactate secretion and the emergence of adipocyte unilocularity (Fig. 1I–K, supplemental Fig. S6B and S7B–E). AMPK ^T172^ phosphorylation correlated significantly with the degree of unilocularity for HUVAS, while no such correlation was seen for multilocular 3D cultures (supplemental Fig. S7D). We thereby conclude increased AMPK activation to coincide with adipocyte unilocularity and lactate production in vitro, and proceeded to investigate this relation further.

Lipid storage in white adipocytes essentially depends on the balance between fatty acid uptake and fatty acid release (lipolysis). AMPK activation is a well-established modulator of adipocyte lipid dynamics, believed to increase basal lipolysis while being mildly inhibitory of isoproterenol-induced lipolysis (40, 41). Accordingly, the key regulatory enzyme for induced lipolysis, hormone-sensitive lipase (HSL), showed reduced activation at both of its major phospho-sites in HUVAS as compared to 2D (for ser660) or 3D (for ser563) (Fig. 6A, B). In line, we also found HUVAS and 3D to be less responsive to lipolytic induction as compared to 2D cultures (Fig. 6C). However, basal lipolysis, most likely more influential on lipid droplet size than induced lipolysis in our steady-state system, was significantly increased for HUVAS and 3D adipocytes (Fig. 6D), in line with their higher AMPK activation. As lipolysis, therefore, could not explain the observed differences in lipid droplet morphology and size between the cultures, we instead turned our focus toward fatty acid uptake. AMPK activation has been shown to increase cellular fatty acid uptake in several other cell types by promoting the translocation of transporter CD36 from intracellular stores to the plasma membrane (42, 43). In accordance, we found unilocular HUVAS adipocytes to display a significantly higher capacity for fatty acid uptake as compared to both 3D and 2D cultures (Fig. 6E). The increased fatty acid uptake was dependent on CD36, as the competitive CD36-inhibitor SMS121 greatly reduced fatty acid uptake into HUVAS adipocytes, but had the opposite effect in the multilocular 3D cultures (44) (Fig. 6F). Notably, while HUVAS adipocytes expressed higher *CD36* mRNA levels than 2D (Fig. 6G), their CD36 protein levels were the lowest among the three cultures, underlining that functional fatty acid uptake tests may be required to correctly assess fatty acid uptake in adipocytes (Fig. 6H and supplemental Fig. S6A). To further confirm the importance of fatty acid uptake for HUVAS lipid droplet size and unilocularity, we chronically treated already differentiated HUVAS adipocytes with CD36-inhibitor SMS121 for 10 days (d20-d30, Fig. 6I). The SMS121-treated spheroids were smaller in size and displayed smaller lipid droplets and greatly reduced unilocularity (Fig. 6I–K and not shown), confirming HUVAS lipid droplet morphology to be highly dependent on fatty acid uptake, similar to white adipocytes in vivo.

**Fig. 7:**
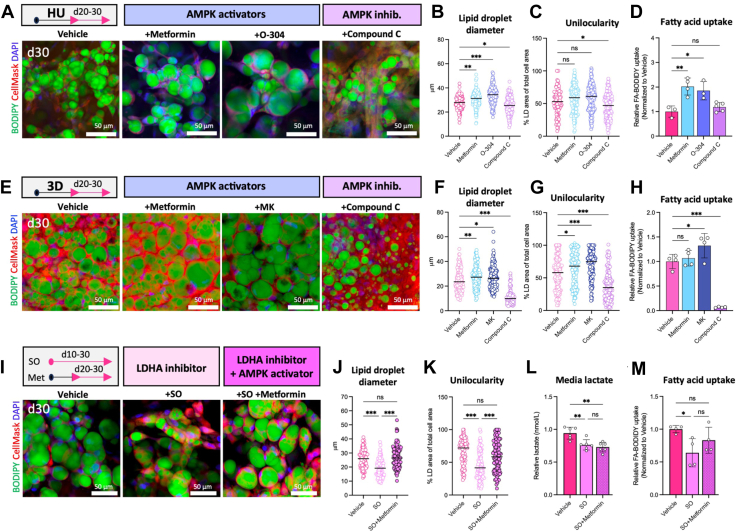
AMPK activation during maturation increases adipocyte fatty acid uptake and unilocularity in vitro. A–D: Representative images (A) as well as quantification of lipid droplet diameter (B), unilocularity (C) and fatty acid uptake (D) of HUVAS adipocytes treated with AMPK agonists metformin and O-304, or with the AMPK inhibitor Compound C from d20 to d30. n = 50 cells per spheroid and 3 spheroids per individual. Scale bars throughout the figure: 50 μm. E–H: Representative images (E) as well as quantification of lipid droplet diameter (F), unilocularity (G) and fatty acid uptake (H) of 3D adipocytes treated with AMPK agonists metformin and MK8722, or AMPK inhibitor Compound C from d20 to d30. n = 50 cells per spheroid and 3 spheroids per individual. I-M: Representative images (I) as well as quantification of lipid droplet diameter (J), unilocularity (K), secreted lactate levels (L), and fatty acid uptake (M) of HUVAS adipocytes treated with aerobic glycolysis inhibitor SO alone (d10-30) or in combination with AMPK agonist metformin (d20-d30). Confocal image quantification was performed on n = 50 cells per spheroid and 2 spheroids per individual.

To corroborate if AMPK activation determines adipocyte lipid morphology in vitro, we treated differentiated HUVAS adipocytes with AMPK modulators. Pharmacological activation of AMPK throughout adipocyte differentiation (d10-d30) with Metformin (41), or treatment with the AMPK inhibitor Compound C replicated previous results from 2D cultures by both impairing HUVAS lipid droplet size and unilocularity independent if AMPK was activated or inhibited (supplemental Fig. S7F–H). We reasoned this was due to the initial adipogenic differentiation phase (d10-20) being less adaptable to metabolic modulation (11). In contrast, treating already differentiated HUVAS adipocytes from d20 onwards with Metformin or the novel pan-AMPK-activator O-304 (45) induced a significant increase in lipid droplet size, larger adipocytes and a trend toward increased unilocularity, while treatment with Compound C had the opposite effect and impaired adipocyte maturation (Fig. 7A–C and supplemental Fig. S7I). AMPK activation with either Metformin or O-304 also increased HUVAS fatty acid uptake from their already high level (Fig. 7D). Moreover, treating multilocular 3D cultures with Metformin or the specific AMPK activator MK8722 significantly increased both lipid droplet diameter, unilocularity and fatty acid uptake, although the functional changed was only seen after MK8722 treatment (Fig. 7E–H). Conversely, Compound C treatment of 3D spheroids from d20 onwards severely impaired lipid droplet formation and adipocyte maturation. Taken together, these data confirm AMPK activation to be able to drive increased lipid droplet size, unilocularity and fatty acid uptake in cultured human white adipocytes.

Finally, we asked if activating AMPK with Metformin can rescue the multilocular phenotype caused by aerobic glycolysis inhibitors. Treating HUVAS spheroids with aerobic glycolysis inhibitor SO throughout differentiation (d10-30) decreased adipocyte lipid droplet size, unilocularity and media lactate levels as observed before, paralleled by a decrease in fatty acid uptake (Fig. 7I–M). However, co-treating the adipocytes with both SO and Metformin during the last 10 days completely rescued the morphological features of HUVAS adipocytes, restoring unilocularity and greatly increasing lipid droplet size (Fig. 7I–K). While Metformin treatment did not affect lactate levels (Fig. 7L), PLIN1 or CIDEC expression (supplemental Fig. S7J, K), it partially restored the defect in fatty acid uptake caused by SO treatment, showing AMPK activation per se to be enough to promote both morphological and functional features of human white adipocytes in HUVAS cultures in vitro (Fig. 7M). Taken together, we conclude that HUVAS unilocularity is determined by a metabolic switch toward aerobic glycolysis, activating AMPK and subsequent CD36-mediated fatty acid uptake, as inhibiting any of these pathways reduces both adipocyte lipid droplet size and the formation of unilocular white adipocytes in vitro.

## Discussion

While no in vitro model can fully recapitulate organ physiology, using the right model remains crucial for dissecting both tissue function and pathology. The previous lack of unilocular adipocyte culture models has limited our understanding of this key aspect of adipocyte biology, and highlights that many fundamental biological adipocyte questions remain only partially answered. By directly comparing three adipocyte culture systems, using isogenic progenitor cells from the same donors, we identify aerobic glycolysis to be the primary cellular program determining adipocyte unilocularity and lipid droplet size in vitro. We use media metabolomics, capillary Western blotting and Seahorse analysis to validate the glycolytic signature of unilocular HUVAS adipocytes, and a range of well-established metabolic inhibitors to show causality. Importantly, promoting aerobic glycolysis increased lipid droplet size via CD36-mediated fatty acid uptake and was enough to achieve unilocularity in otherwise multilocular 3D-cultures. Furthermore, we found that AMPK activation could rescue defective aerobic glycolysis and fatty acid uptake, while elevated lactate production did not induce UCP1 expression or HIF1α stabilization, in contrast to earlier observations (35, 46, 47). Together, our results highlight the large differences in culture conditions can infer and suggest that unilocular in vitro differentiated adipocytes may resemble their in vivo counterparts more closely both morphologically and functionally.

3D cultures have recently emerged as superior to traditional 2D systems by more accurately reproducing the morphological and transcriptional features of primary adipocytes, owing to their enhanced tissue-like microenvironment (32). However, as our study demonstrates, not all 3D models are equivalent, with substantial differences in adipocyte morphology, expression, metabolism and fatty acid handling between different 3D models. Surprisingly, unilocular HUVAS adipocytes did not express higher protein levels of canonical adipocyte markers such as PPARγ, ATGL, or FAS compared to their multilocular 3D and 2D counterparts (Fig. 1G). This may reflect the known autoregulatory suppression of PPARγ under high activation conditions (48, 49, 50), observed in this study during later adipocyte maturation (d20-d30, Fig. 1L–O), which would also dampen expression of downstream targets such as ATGL. In addition, our commercially available culture media does not include thiazolidinediones, potentially allowing this autoregulatory downregulation to occur during adipocyte maturation. The results nonetheless underline that while classical adipogenic markers are reliable indicators when comparing adipocytes to non-adipocytes (28), their expression levels may not correlate with adipocyte maturity or unilocularity when comparing only adipocytes among each other. This would explain why we found few canonical adipocyte markers among the top genes correlating to lipid droplet size in our dataset, and it highlights the need for definition of novel markers that could be used to discern the degree of adipocyte maturation and unilocularity from transcriptional data. We hypothesize that such markers would be useful when studying the lipid storage capacity of subcutaneous adipose tissue and could be used to screen for pharmacological modulators that enhance safe lipid storage.

Rather than canonical adipocyte markers, we found glycolytic and metabolic pathways to be most strongly correlated with lipid droplet size and unilocularity in vitro. Similar to many other proliferative or differentiating cell types, adipocytes are well known to convert a substantial amount of their mobilized glucose into lactate—a prioritized metabolic feature for adipocytes that correlates with both their size and triglyceride content (37, 51, 52). However, previous tracing studies of differentiating multilocular 2D adipocytes has indicated lactate production to be important mainly during early adipogenic differentiation, the opposite to our results, whereafter it becomes downregulated as mitochondrial glucose oxidation becomes the dominant pathway instead (12, 34). We speculate that the high need for de novo lipogenesis by 2D cultures (10), which requires mitochondrial substrate oxidation, may prevent them from fully converting to relying on aerobic glycolysis after differentiation (12). This would explain why pharmacological OXPHOS inhibitors previously have been shown to *reduce* lipid droplet size in 2D cultured adipocytes (11), while we observed increased lipid content in the HUVAS adipocytes that rely mainly on aerobic glycolysis (Fig. 5). In further support, neither FOXP1 nor PDK2 overexpression in 2D adipocytes, despite both being strong promoters of aerobic glycolysis, was sufficient to induce unilocularity (35, 53), again contrasting our findings where PDK2 is more highly expressed in HUVAS. In contrast, PDK2 knockout in vivo in obese mice, inhibiting aerobic glycolysis via a similar mechanism as PB used in this study, significantly reduced adipocyte lipid droplet size and mouse body weight (35). These results provide proof of principle that manipulating aerobic glycolysis in unilocular adipocytes in vivo affects their lipid content and storage capacity in the same fashion as observed here, although not to the same extent as in vitro as the adipocytes lacking PDK2 remained unilocular. It also underlines the recent surge in interest regarding adipocyte metabolism (54, 55) and the need for further understanding of how culture conditions affect the results we gain.

Our comparative analysis across three isogenic culture models identified AMPK activation to be central for connecting adipocyte metabolism, unilocularity and fatty acid uptake in 3D cultures (Figs. 6 and 7). Pharmacological AMPK activation with either Metformin, O-304 or MK8722 (41, 45) during adipocyte maturation enlarged adipocyte lipid droplets and promoted unilocularity in both 3D and HUVAS cultures, while its inhibition had the opposite effect. This is in part in line with reports from iPSC-derived adipocyte 3D organoids, which take up and accumulate more fatty acid-tracers in response to Metformin treatment (56), but contrasts previous reports from mice and 2D cultures. In rodents, AMPK activation by AICAR has been shown to promote weight loss, and in 2D cultures it induces multilocularity, beiging, and enhanced the degradation the lipid transferase CIDEC, thereby reducing lipid droplet fusion and unilocularity (23, 57, 58). The differences in vivo are well established to be due to AICAR acting on multiple organ systems, including liver and hypothalamus. We propose that the contrasting results from 2D cultured adipocytes stem from most interventions focused on AICAR and AMPK activation study only their effects on early adipogenic differentiation (57), rather than the later maturation phase or ready formed adipocytes as in this study (d20-30, Fig. 7). Other determining factors for adipocyte AMPK activation may be differences in mitochondrial content between 2D cultures and HUVAS (Fig. 3E, F), their distinct microenvironments or the high pro-inflammatory transcription pattern of 2D cells reported here (Fig. 2G) (31). Indeed, ∼90% of the transcriptomic variance in our study was explained by culture dimensionality (Fig. 2B), and 2D adipocytes remained more similar to preadipocytes than mature adipocytes both in terms of adipogenic marker expression and metabolic profile (Fig. 2H–J), potentially reflecting the divergent responses to metabolic inhibitors between 2D and 3D adipocyte cultures. The stiffness of the extracellular environment is well known to affect cell signaling and metabolism, among others by increased stiffness and subsequent YAP/TAZ nuclear localization activating aerobic glycolysis in cancer cells (59). However, further studies are clearly needed to delineate the interplay between culture microenvironment, metabolism and adipocyte differentiation and function.

HUVAS spheroids, while larger than standard 3D spheroids (Fig. 1), are more porous due to adipocyte differentiation along vascular sprouts embedded in the Matrigel scaffold (4, 17), likely preventing hypoxia. Our data suggest that the transcriptional enrichment for hypoxia-related GO terms in HUVAS (Fig. 2D, E) reflects shared signatures between aerobic glycolysis and hypoxia rather than true oxygen deprivation. In support, we observed no HIF1α stabilization in HUVAS cultures despite sustained high lactate levels, in contrast to previous reports for 2D cultures (35). Moreover, canonical hypoxia-induced genes such as *HIF1A, ARNT, VEGFC, and TGFB1* were more highly expressed in 2D than in HUVAS cells (Fig. 4 and data not shown). Notably, high lactate production did not induce UCP1 expression or beiging in HUVAS adipocytes either (Fig. 1G), further emphasizing the divergence in metabolic signaling between different culture systems (46, 47). Although lactate can activate GPR81 and suppress lipolysis (60), the high basal lipolysis rates in both HUVAS and 3D spheroids (Fig. 6D) argue against active GPR81 signaling driving the HUVAS phenotype. We propose instead that the persistently elevated extracellular lactate levels may drive GPR81 internalization and potentially further contribute to fueling the high lipolytic activity observed in 3D cultures (60).

Finally, it should be noted that while increased adipocyte size and lipid droplet enlargement are traditionally associated with metabolic dysfunction, growing evidence indicates that *impaired* lipid storage in subcutaneous fat depots may be a key contributor to disease development (1, 2). The increased lipid droplet size observed in HUVAS in response to Rotenone and Metformin treatment may be seen as symptoms of higher favorable fatty acid uptake, promoting healthy lipid storage rather than mimicking the hypertrophic state of lipid overloading that occurs in obesity, in line with Metformin’s beneficial effects on systemic metabolism. As AMPK activation inhibits adipocyte de novo lipogenesis (40) in parallel to inducing CD36 translocation and fatty acid uptake (42, 43), AMPK will promote cultured adipocytes to take up, rather than synthesize, their triglycerides, highlighting adipocyte fatty acid uptake as an important physiological cell program. Likewise, inhibiting unilocularity in vivo by genetic deletion or mutations in CIDEC causes insulin resistance and diabetes in both humans and mice (9, 19). While thiazolidinediones promoting subcutaneous adipocyte lipid storage did not pan out as an effective therapeutic avenue in the end, they provided further proof of concept that increasing safe lipid storage could serve as a therapeutic strategy in the future. However, whether the HUVAS model can inform such efforts and ensure increased research translatability in the future remains to be determined.

Taken together, our findings highlight the critical role of culture conditions in shaping adipocyte metabolism and morphology in vitro. We define a triad of aerobic glycolysis, AMPK activation and fatty acid uptake as key determinants of lipid droplet size and unilocularity and demonstrate that different 3D models exhibit distinct metabolic and functional phenotypes. Our results offer mechanistic insights into how adipocytes regulate triglyceride storage and provide a foundation for future refinement of adipocyte culture systems that better model human adipose biology and lipid storage in health and disease.

## Data availability

The data that support the findings of this study are available from the corresponding author upon reasonable request.

## Supplemental data

This article contains supplemental data.

## Conflict of interest

The authors declare that they do not have any conflicts of interest with the content of this article.
