# Supplementary material for: Aerobic glycolysis drives differentiation of unilocular adipocytes
Source: J Lipid Res. 2026 Mar 17;67(4):101023. doi: 10.1016/j.jlr.2026.101023 (PMC13091052; doi:10.1016/j.jlr.2026.101023)
Supplement: Supplemental data [file mmc1.pdf]

Supplemental data

Aerobic glycolysis drives differentiation of unilocular adipocytes

Alice Maestri, Min Cai*, Ruby Schipper*, Julia Backman, Alana Vannay, Anneli Olsson, Ewa Ehrenborg, Roland Nilsson, Carolina E Hagberg.

Suppl. Fig. S1

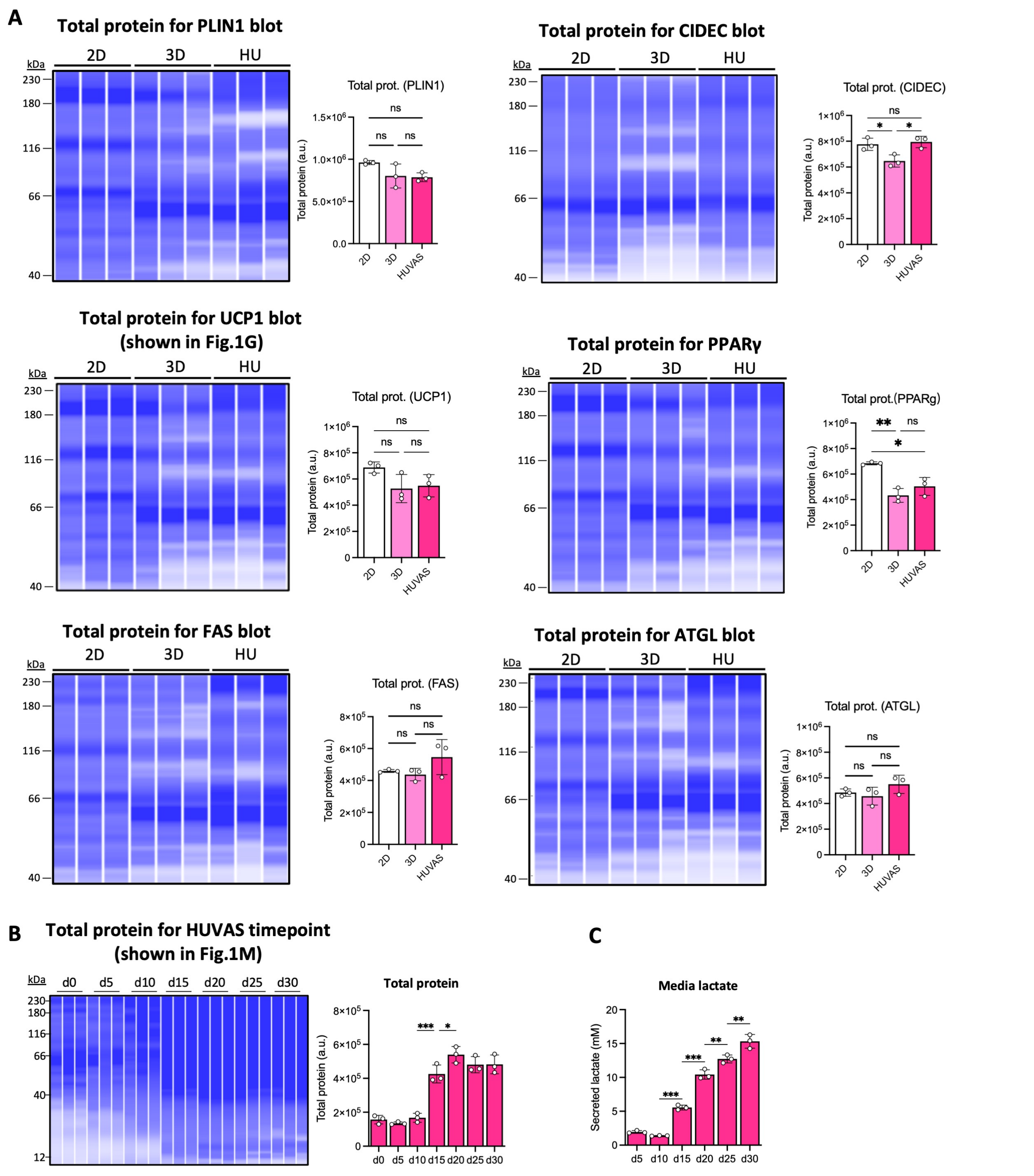

A
Total protein for PLIN1 blot
kDa
2D
3D
HU
230
180
116
66
40
Total prot. (PLIN1)
Total protein (a.u.)
1.5×10^6
1.0×10^6
5.0×10^5
0.0
ns
2D
3D
HUVAS
Total protein for CIDEC blot
Total prot. (CIDEC)
1×10^6
8×10^5
6×10^5
4×10^5
2×10^5
0
*
Total protein for UCP1 blot
(shown in Fig.1G)
Total prot. (UCP1)
Total protein for PPARγ
Total prot.(PPARg)
**
Total protein for FAS blot
Total prot. (FAS)
Total protein for ATGL blot
Total prot. (ATGL)
B
Total protein for HUVAS timepoint
(shown in Fig.1M)
d0
d5
d10
d15
d20
d25
d30
12
Total protein
***
C
Media lactate
Secreted lactate (mM)
20
15
10
5

Suppl. Fig. S2

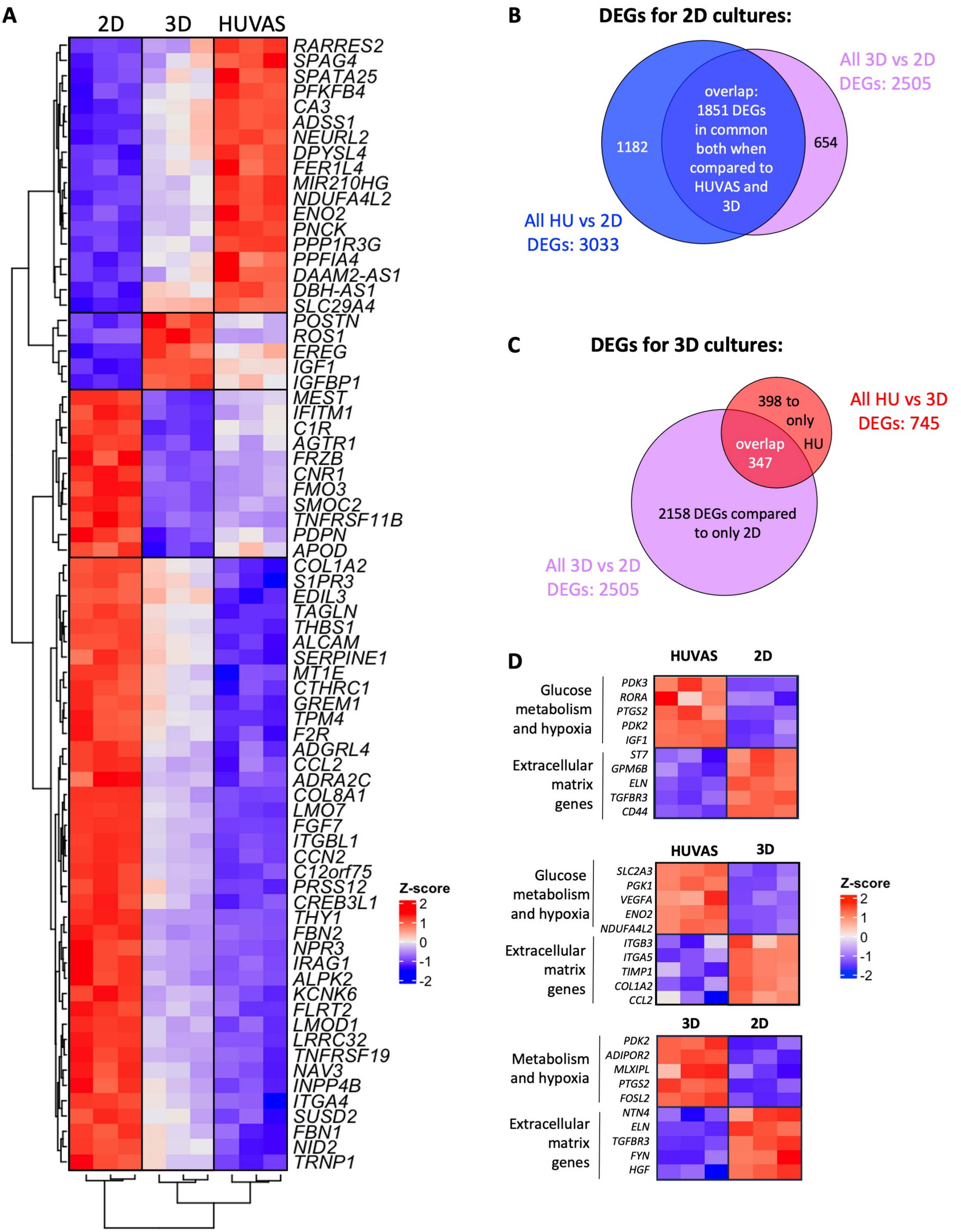

A
2D
3D
HUVAS
RARRES2
SPAG4
SPATA25
PFKFB4
CA3
ADSS1
NEURL2
DPYSL4
FER1L4
MIR210HG
NDUFA4L2
ENO2
PNCK
PPP1R3G
PPFIA4
DAAM2-AS1
DBH-AS1
SLC29A4
POSTN
ROS1
EREG
IGF1
IGFBP1
MEST
IFITM1
C1R
AGTR1
FRZB
CNR1
FMO3
SMOC2
TNFRSF11B
PDPN
APOD
COL1A2
S1PR3
EDIL3
TAGLN
THBS1
ALCAM
SERPINE1
MT1E
CTHRC1
GREM1
TPM4
F2R
ADGRL4
CCL2
ADRA2C
COL8A1
LMO7
FGF7
ITGBL1
CCN2
C12orf75
PRSS12
CREB3L1
THY1
FBN2
NPR3
IRAG1
ALPK2
KCNK6
FLRT2
LMOD1
LRRC32
TNFRSF19
NAV3
INPP4B
ITGA4
SUSD2
FBN1
NID2
TRNP1
Z-score
2
1
0
-1
-2
B
DEGs for 2D cultures:
All 3D vs 2D DEGs: 2505
overlap: 1851 DEGs in common both when compared to HUVAS and 3D
1182
654
All HU vs 2D DEGs: 3033
C
DEGs for 3D cultures:
398 to only HU
All HU vs 3D DEGs: 745
overlap 347
2158 DEGs compared to only 2D
All 3D vs 2D DEGs: 2505
D
HUVAS
2D
Glucose metabolism and hypoxia
PDK3
RORA
PTGS2
PDK2
IGF1
Extracellular matrix genes
ST7
GPM6B
ELN
TGFBR3
CD44
HUVAS
3D
Glucose metabolism and hypoxia
SLC2A3
PGK1
VEGFA
ENO2
NDUFA4L2
Extracellular matrix genes
ITGB3
ITGA5
TIMP1
COL1A2
CCL2
Z-score
2
1
0
-1
-2
3D
2D
Metabolism and hypoxia
PDK2
ADIPOR2
MLXIPL
PTGS2
FOSL2
Extracellular matrix genes
NTN4
ELN
TGFBR3
FYN
HGF

Suppl. Fig. S3

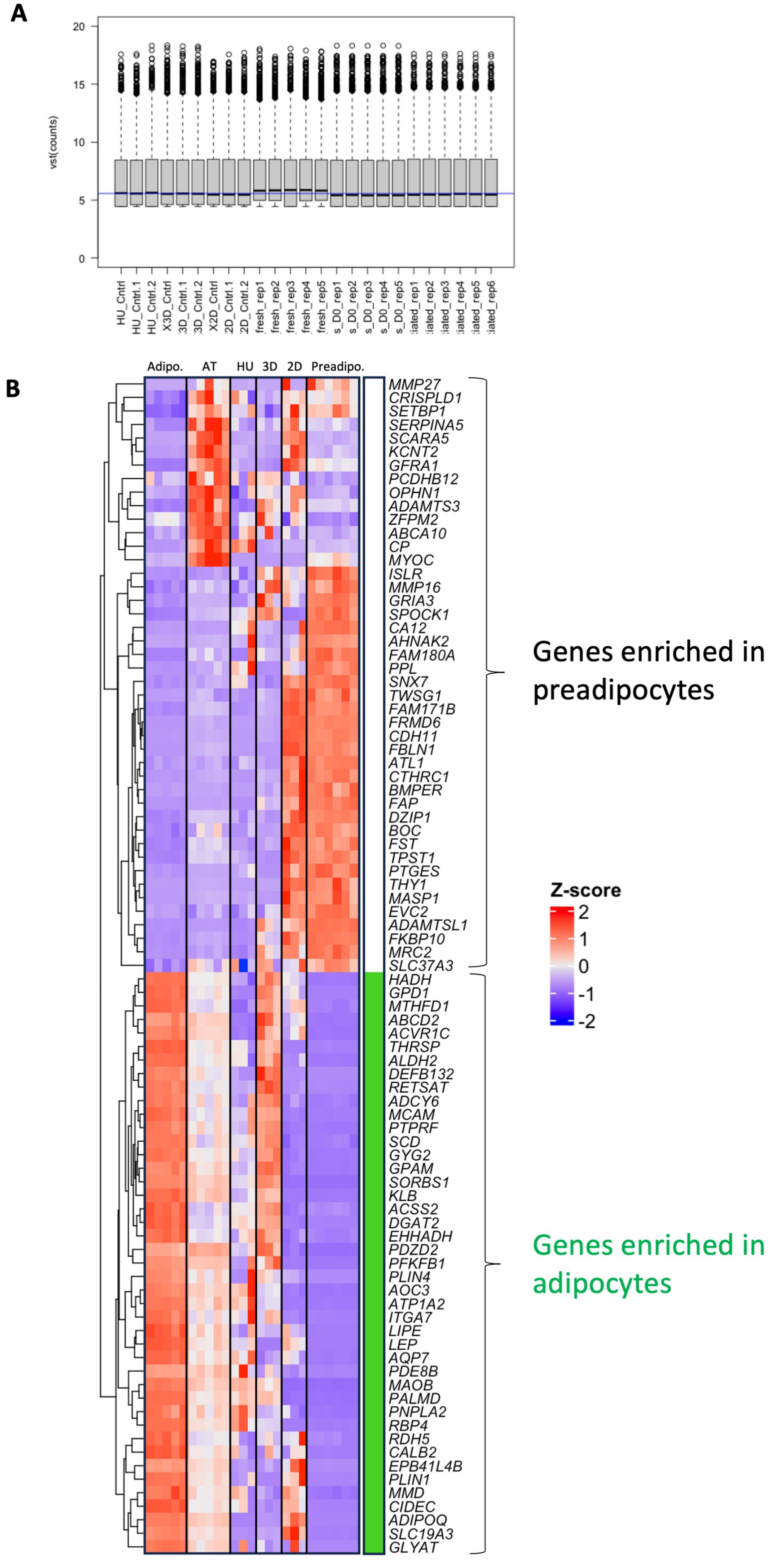
A
20
15
10
5
0
vst(counts)
HU_Cntrl
HU_Cntrl.1
HU_Cntrl.2
X3D_Cntrl
3D_Cntrl.1
3D_Cntrl.2
X2D_Cntrl
2D_Cntrl.1
2D_Cntrl.2
fresh_rep1
fresh_rep2
fresh_rep3
fresh_rep4
fresh_rep5
s_D0_rep1
s_D0_rep2
s_D0_rep3
s_D0_rep4
s_D0_rep5
iated_rep1
iated_rep2
iated_rep3
iated_rep4
iated_rep5
iated_rep6
B
Adipo.
AT
HU
3D
2D
Preadipo.
MMP27
CRISPLD1
SETBP1
SERPINA5
SCARA5
KCNT2
GFRA1
PCDHB12
OPHN1
ADAMTS3
ZFPM2
ABCA10
CP
MYOC
ISLR
MMP16
GRIA3
SPOCK1
CA12
AHNAK2
FAM180A
PPL
SNX7
TWSG1
FAM171B
FRMD6
CDH11
FBLN1
ATL1
CTHRC1
BMPER
FAP
DZIP1
BOC
FST
TPST1
PTGES
THY1
MASP1
EVC2
ADAMTSL1
FKBP10
MRC2
SLC37A3
HADH
GPD1
MTHFD1
ABCD2
ACVR1C
THRSP
ALDH2
DEFB132
RETSAT
ADCY6
MCAM
PTPRF
SCD
GYG2
GPAM
SORBS1
KLB
ACSS2
DGAT2
EHHADH
PDZD2
PFKFB1
PLIN4
AOC3
ATP1A2
ITGA7
LIPE
LEP
AQP7
PDE8B
MAOB
PALMD
PNPLA2
RBP4
RDH5
CALB2
EPB41L4B
PLIN1
MMD
CIDEC
ADIPOQ
SLC19A3
GLYAT
Genes enriched in preadipocytes
Z-score
2
1
0
-1
-2
Genes enriched in adipocytes

Suppl. Fig. S4

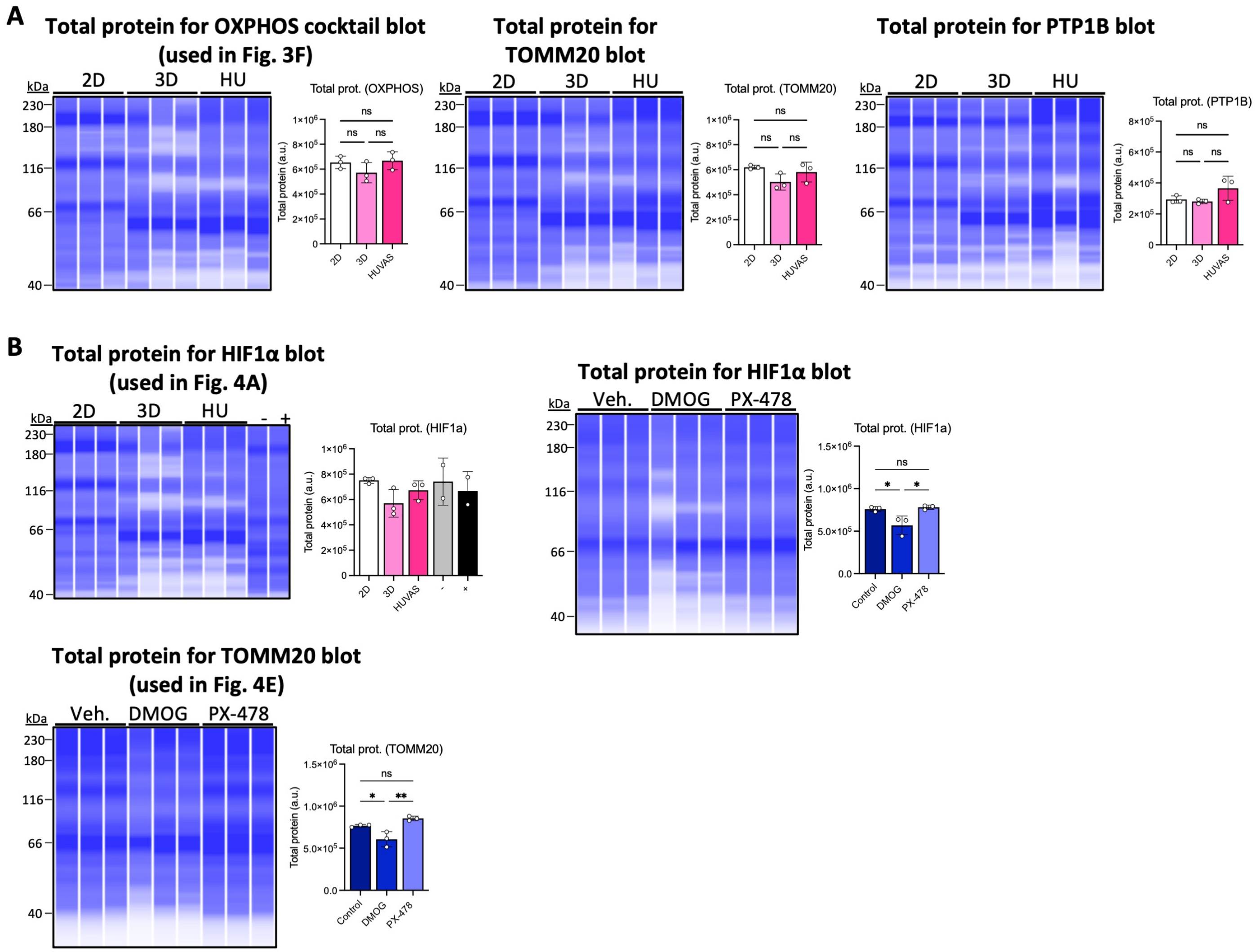
A
Total protein for OXPHOS cocktail blot (used in Fig. 3F)
kDa
2D
3D
HU
230
180
116
66
40
Total prot. (OXPHOS)
Total protein (a.u.)
ns
2D
3D
HUVAS
Total protein for TOMM20 blot
Total prot. (TOMM20)
Total protein for PTP1B blot
Total prot. (PTP1B)
B
Total protein for HIF1α blot (used in Fig. 4A)
-
+
Total prot. (HIF1a)
Total protein for HIF1α blot
Veh.
DMOG
PX-478
Control
*
Total protein for TOMM20 blot (used in Fig. 4E)
**

Suppl. Fig. S5

A

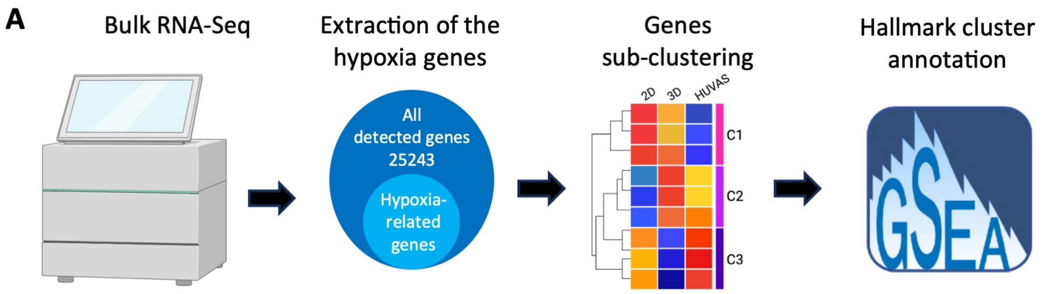

B Clustering all major hypoxia-associated genes based on GO:0001666

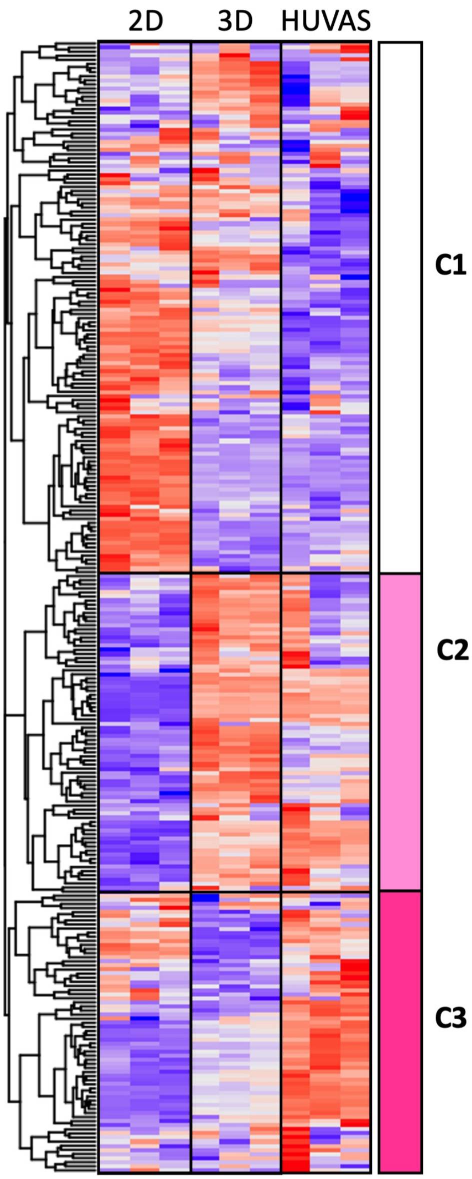

C1

Gene Set Name [# Genes (K)]	# Genes in Overlap (k)	p-value	FDRq-value
Epithelial-mesenchymal transition [200]	11	$3.33 e^{-11}$	$1.66 e^{-9}$
Coagulation [138]	9	$4.79 e^{-10}$	$1.2 e^{-8}$
Apoptosis [161]	8	$3.86 e^{-8}$	$6.43 e^{-7}$
TGF beta signaling [54]	5	$6.89 e^{-7}$	$8.61 e^{-6}$
Allograft rejection [200]	7	$2.95 e^{-6}$	$1.64 e^{-5}$
Complement [200]	7	$2.95 e^{-6}$	$1.64 e^{-5}$
Hypoxia [200]	7	$2.95 e^{-6}$	$1.64 e^{-5}$
P53 pathway [200]	7	$2.95 e^{-6}$	$1.64 e^{-5}$
TNFA signaling via NFKB [200]	7	$2.95 e^{-6}$	$1.64 e^{-5}$
Apical junction [200]	6	$3.66 e^{-5}$	$1.31 e^{-4}$

C2

Gene Set Name [# Genes (K)]	# Genes in Overlap (k)	p-value	FDRq-value
Adipogenesis [200]	8	$3.64 e^{-9}$	$9.11 e^{-8}$
Hypoxia [200]	8	$3.64 e^{-9}$	$9.11 e^{-8}$
Apoptosis [161]	6	$5.46 e^{-7}$	$9.1 e^{-6}$
MTOC1 signaling [200]	6	$1.93 e^{-6}$	$2.41 e^{-5}$
NOTCH signaling [32]	3	$2.82 e^{-5}$	$1.93 e^{-4}$
Glycolysis [200]	5	$3.48 e^{-5}$	$1.93 e^{-4}$
P53 pathway [200]	5	$3.48 e^{-5}$	$1.93 e^{-4}$
TNFA signaling via NFKB [200]	5	$3.48 e^{-5}$	$1.93 e^{-4}$
Xenobiotic metabolism [200]	5	$3.48 e^{-5}$	$1.93 e^{-4}$
PI3K AKT MTOR signaling [105]	4	$4.33 e^{-5}$	$2.16 e^{-4}$

C3

Gene Set Name [# Genes (K)]	# Genes in Overlap (k)	p-value	FDRq-value
Hypoxia [200]	18	$5.88 e^{-27}$	$2.94 e^{-25}$
Glycolysis [200]	13	$9.14 e^{-18}$	$2.28 e^{-16}$
MTOC1 signaling [200]	8	$1.2 e^{-9}$	$1.99 e^{-8}$
P53 pathway [200]	6	$8.56 e^{-7}$	$8.56 e^{-6}$
TNFA signaling via NFKB [200]	6	$8.56 e^{-7}$	$8.56 e^{-6}$
KRAS signaling up [200]	5	$1.78 e^{-5}$	$1.49 e^{-4}$
Unfolded protein response [113]	4	$3.36 e^{-5}$	$2.4 e^{-4}$
Apoptosis [161]	4	$1.33 e^{-4}$	$8.3 e^{-4}$
IL2 STAT5 signaling [199]	4	$2.98 e^{-4}$	$1.38 e^{-3}$
E2F targets [200]	4	$3.04 e^{-4}$	$1.38 e^{-3}$

Suppl. Fig. S6

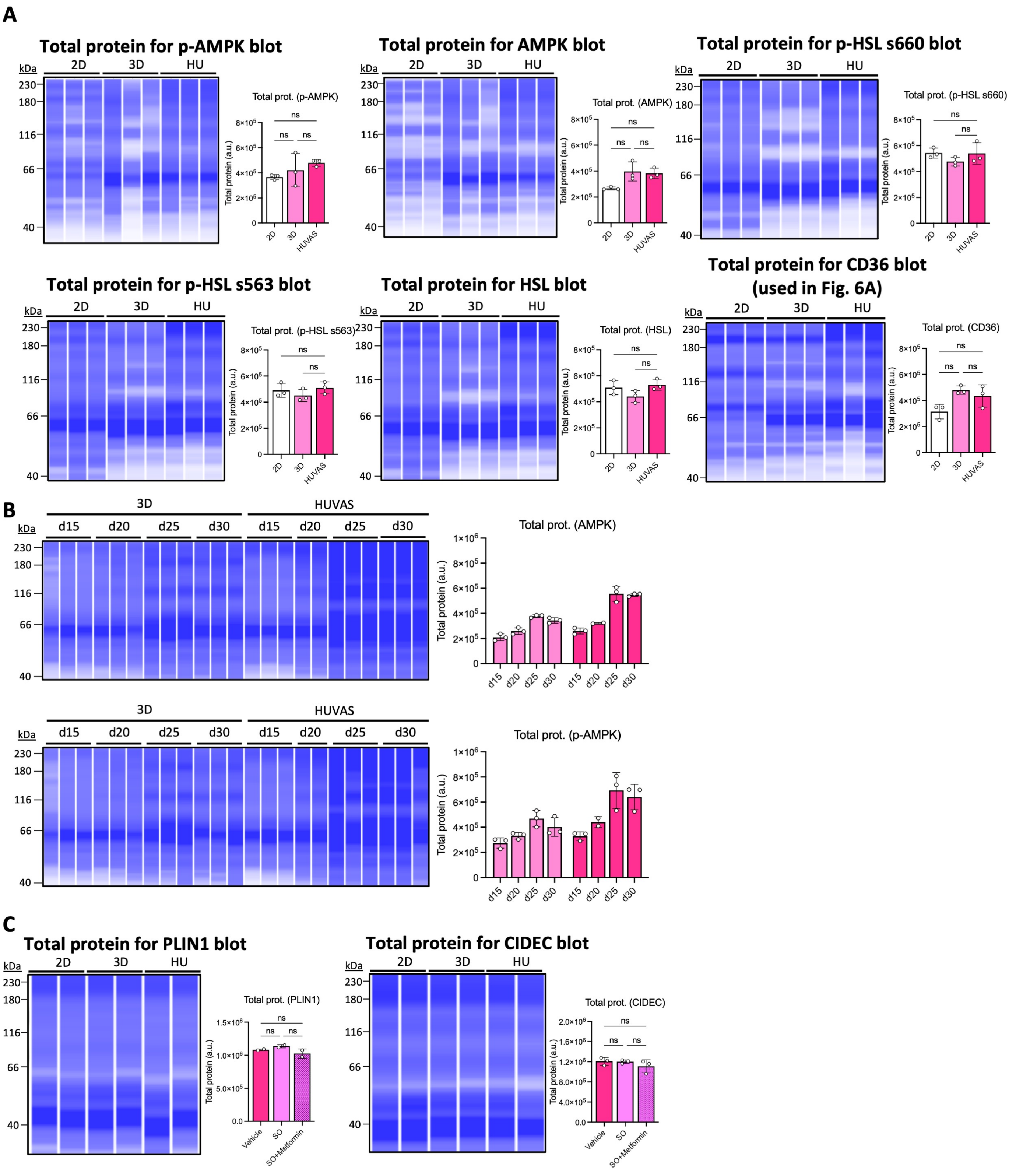
A
Total protein for p-AMPK blot
Total prot. (p-AMPK)
Total protein for AMPK blot
Total prot. (AMPK)
Total protein for p-HSL s660 blot
Total prot. (p-HSL s660)
Total protein for p-HSL s563 blot
Total prot. (p-HSL s563)
Total protein for HSL blot
Total prot. (HSL)
Total protein for CD36 blot
(used in Fig. 6A)
Total prot. (CD36)
kDa
2D
3D
HU
HUVAS
Total protein (a.u.)
ns
B
3D
HUVAS
d15
d20
d25
d30
Total prot. (AMPK)
Total prot. (p-AMPK)
C
Total protein for PLIN1 blot
Total prot. (PLIN1)
Total protein for CIDEC blot
Total prot. (CIDEC)
Vehicle
SO
SO+Metformin

Suppl. Fig. S7

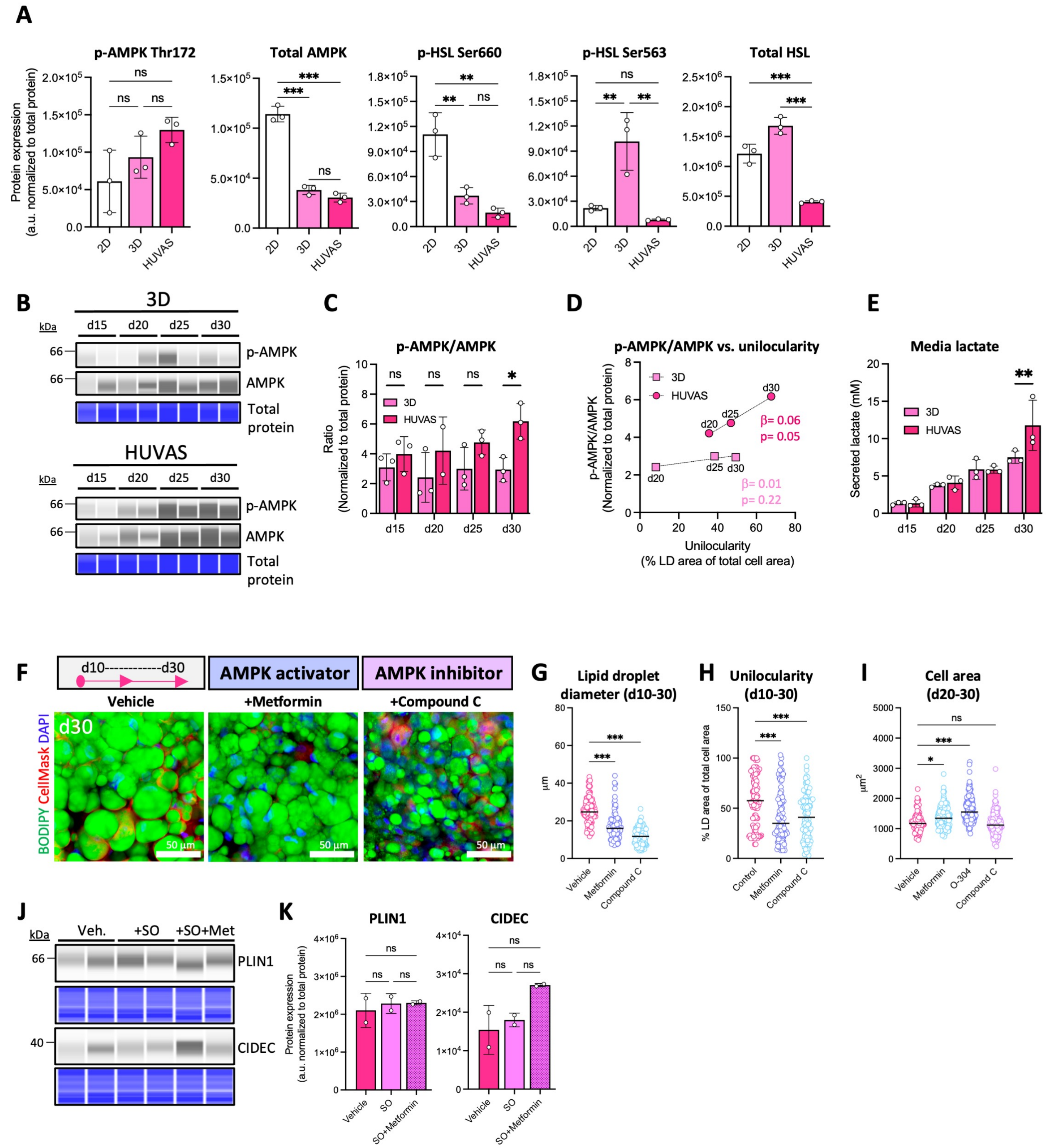

A
p-AMPK Thr172
Total AMPK
p-HSL Ser660
p-HSL Ser563
Total HSL
Protein expression
(a.u. normalized to total protein)
2D
3D
HUVAS
ns
***
**
B
3D
HUVAS
kDa
d15
d20
d25
d30
66
p-AMPK
AMPK
Total protein
C
p-AMPK/AMPK
Ratio
(Normalized to total protein)
D
p-AMPK/AMPK vs. unilocularity
p-AMPK/AMPK
(Normalized to total protein)
β= 0.06
p= 0.05
β= 0.01
p= 0.22
Unilocularity
(% LD area of total cell area)
E
Media lactate
Secreted lactate (mM)
F
d10------------d30
AMPK activator
AMPK inhibitor
Vehicle
+Metformin
+Compound C
BODIPY CellMask DAPI
d30
50 µm
G
Lipid droplet diameter (d10-30)
µm
H
Unilocularity (d10-30)
% LD area of total cell area
I
Cell area (d20-30)
µm²
Vehicle
Control
Metformin
Compound C
O-304
J
Veh.
+SO
+SO+Met
40
PLIN1
CIDEC
K
PLIN1
CIDEC
Vehicle
SO
SO+Metformin
